# Molecular mechanism of selective substrate engagement and inhibitor disengagement of cysteine synthase

**DOI:** 10.1074/jbc.RA120.014490

**Published:** 2020-11-24

**Authors:** Abhishek Kaushik, R. Rahisuddin, Neha Saini, Ravi P. Singh, Rajveer Kaur, Sukirte Koul, S. Kumaran

**Affiliations:** G. N. Ramachandran Protein Center, Institute of Microbial Technology (IMTECH), Council of Scientific and Industrial Research (CSIR), Sector 39-A, Chandigarh, India

**Keywords:** competitive allostery, substrate selectivity, cysteine synthesis, X-ray crystallography, fluorescence, CD, circular dichroism, CRC, cysteine regulatory complex, CS, cysteine synthase, OASS, O-acetyl serine sulfhydrylase, SAT, serine acetyltransferase

## Abstract

O-acetyl serine sulfhydrylase (OASS), referred to as cysteine synthase (CS), synthesizes cysteine from O-acetyl serine (OAS) and sulfur in bacteria and plants. The inherent challenge for CS is to overcome 4 to 6 log-folds stronger affinity for its natural inhibitor, serine acetyltransferase (SAT), as compared with its affinity for substrate, OAS. Our recent study showed that CS employs a novel competitive-allosteric mechanism to selectively recruit its substrate in the presence of natural inhibitor. In this study, we trace the molecular features that control selective substrate recruitment. To generalize our findings, we used CS from three different bacteria (*Haemophilus*, *Salmonella*, and *Mycobacterium*) as our model systems and analyzed structural and substrate-binding features of wild-type CS and its ∼13 mutants. Results show that CS uses a noncatalytic residue, M120, located 20 Å away from the reaction center, to discriminate in favor of substrate. M120A and background mutants display significantly reduced substrate binding, catalytic efficiency, and inhibitor binding. Results shows that M120 favors the substrate binding by selectively enhancing the affinity for the substrate and disengaging the inhibitor by 20 to 286 and 5- to 3-folds, respectively. Together, M120 confers a net discriminative force in favor of substrate by 100- to 858-folds.

Enzymes inside the cell have to bind their substrates selectively, discriminating against a large number of molecules that may mimic substrates (1, 2). Substrate selection and ligand discrimination mechanisms have been studied for a variety of enzymes (3, 4). In general, two major mechanisms of ligand recognitions, conformational selection and induced-fit, have been studied well (5). A number of studies have reported that enzymes employ induced-fit mechanism to form catalytically competent “enzyme·substrate complex” as compared with short-lived, nonproductive “enzyme·competitor·ligand” complexes (6, 7). Because substrate selectivity is a very important property of catalytic activity, active site features of enzymes act like filters to screen and select the cognate substrate (8, 9). Many enzymes, proteases in particular, have to select their substrates in the presence of natural inhibitors (10, 11). Proteases accomplish substrate specificity through specific subpockets (S1, S2), mostly noncatalytic residues, distributed within the active site (9, 12). Similarly, the active site of cysteine synthesis enzyme (CS) is mapped into three subpockets (S1, S2, S3) that coordinate the substrate-induced conformational transition to closed state (13). In a recent study, we showed that substrate-binding-induced active site conformational changes allowed CS to selectively recruit its substrate, O-acetyl serine (OAS), in the presence of high-affinity natural inhibitor, the C-terminal of serine acetyl transferase (SAT). Our extensive structural and analytical work revealed that only substrate binding can induce the active site of CS to a closed state, not the binding of the high-affinity inhibitor. We proposed a novel “competitive-allosteric” mechanism by which CS selectively recruits its substrate while bound to high-affinity natural inhibitors (13). In this work, we traced the molecular origins of substrate selectivity by employing a combination of approaches and provide evidences for substrate selectivity.

CS and its natural inhibitor, SAT, catalyze the last two steps of cysteine synthesis (14). Further, both interact to form a stable multienzyme complex, referred to as cysteine regulatory complex (CRC) (15, 16, 17). Our recent studies showed that CRC complex dissociates in the presence of OAS, the substrate of CS, at stoichiometric concentrations (13, 18). Dissociation of CRC at stoichiometric concentration of OAS was not expected as previous studies reported that affinity of SAT (inhibitor) is 4 to 6 log-fold higher than that of OAS (14, 15, 16, 17, 18). Binary and ternary complexes of enzyme·substrate, enzyme·inhibitor, and enzyme·inhibitor·substrate complexes, in combination with fast kinetics, showed that CS achieves selective substrate recruitment in the presence of high-affinity inhibitor by employing a novel “competitive-allosteric” mechanism (13). Extensive structural and biochemical work shows that structures captured with substrate bound to the active site have substrate-induced conformational switch to a closed state (19). Glucose-binding-induced ATPase activity of hexokinase, steric switch mechanism of ribosome, correct substrate base-binding-induced organization of DNA polymerase active site are classical examples of substrate-induced functional specificity (20, 21, 22, 23, 24). However, mechanism of substrate selectivity achieved during cysteine synthesis step by CS is different because, the active site-bound high-affinity inhibitor must be removed before substrate enters.

The most important difference is the mode of recognition of inhibitor and substrate by CS (25). Although the physiological significance of CRC is not yet known, our recent study established the connection between induced-fit structural change and selective recruitment of the substrate (13). We traced the molecular origins of substrate selectivity in this study. We analyzed structural, activity, and ligand recognition properties of CS from three different species (*Haemophilus*, *Salmonella*, and *Mycobacterium*) and ∼13 CS mutants of these three enzymes. Our results show that mutation of either M120 or M92 significantly reduces the substrate binding and catalytic efficiency of CS, whereas M96 shows limited or insignificant contribution to substrate recruitment. We present a generalized model of gated substrate-recruitment mechanism in which M120 acts as a gate sensor, which triggers allosteric conformational changes that disengage inhibitor (SAT) and allow OAS to enter.

## Results

### Analyses of active site channel features for identification of substrate discriminative residues

**Figure 1:**
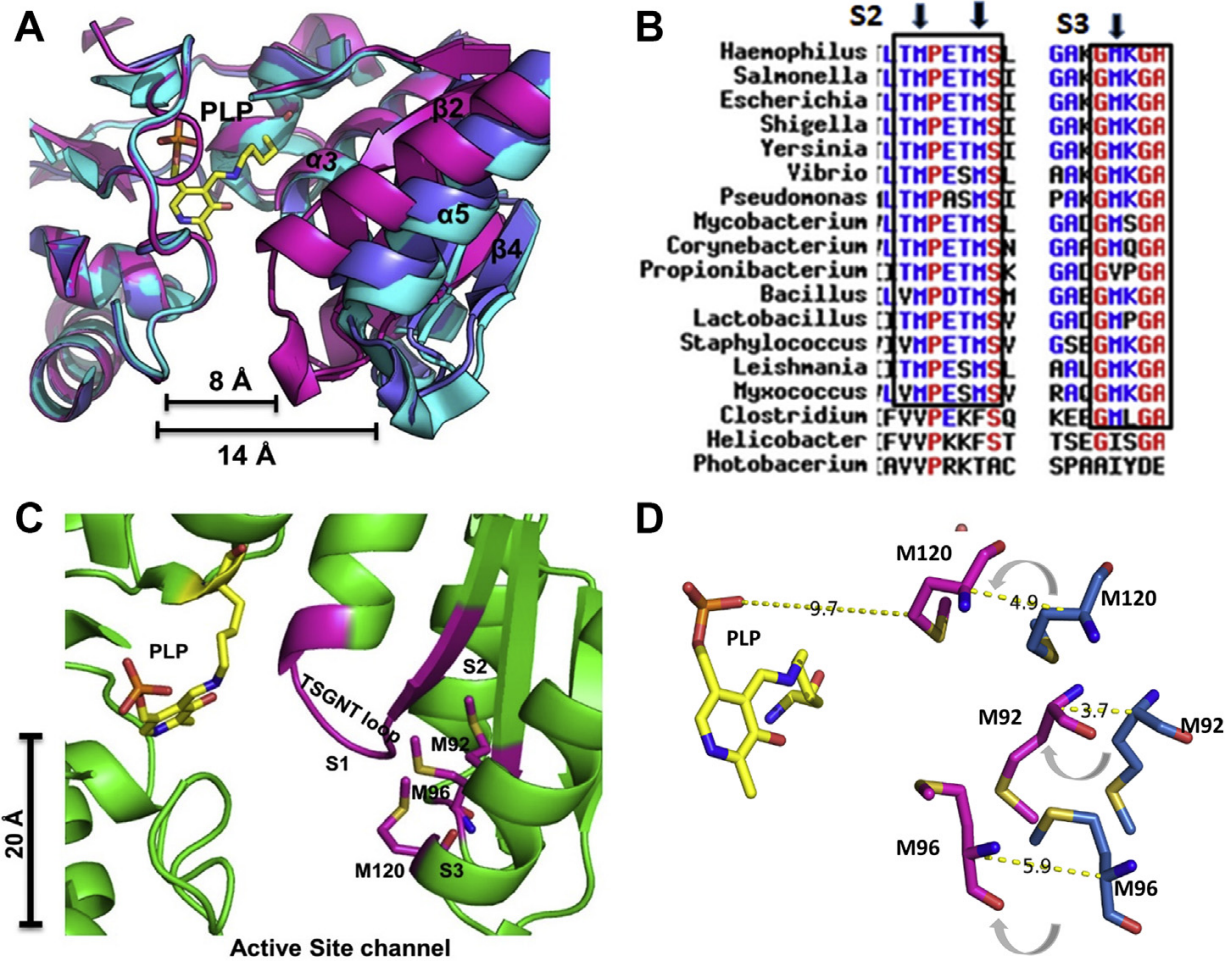
**The general feature of movable domain in CS.***A*, the *Hi*CS apo form of CS (blue, PDB ID 4HO1) is superposed with *Hi*CS-inhibitor-bound (Cyan, PDB ID 1Y7L) and substrate-bound closed form of *Hi*CS (*magenta*, PDB ID: 5DBH). Distance measurements show that both apo and inhibitor-bound forms are in the open state, and the active site entrance width of the closed state is reduced by 8 Å. *B*, a view of multiple sequence alignment (MSA) of CS sequences from different organisms. The selected window region is in alignment with structurally mapped subsites (S2 and S3) of active site entrance. Conserved methionine residues are highlighted. *C*, CS active-site architecture with three subsites (S1, S2, and S3), which are numbered to map their role in the catalytic cycle; S1—site of reaction (residues ^69^TSGNT^73^ loop), S2—substrate translocation (M92, M96), S3—substrate binding/recruitment (M120). PLP bound to K42 marks the reaction center. *D*, relative positional locations of three residues (M120, M96, and M92) in the movable domain are shown. Distance between M120 in the closed state and active site PLP is highlighted. In the closed state, M120 is 9.7 Å away from PLP.

CS from both bacteria and plants switch to closed-state structure only upon binding to the substrate, not the inhibitor, SAT C-terminal. The substrate-induced structural change is of very significant magnitude, evidenced by multiple crystal structures of CS in complex with OAS (26,27). As shown in the Figure 1*A*, the α-helix5 moves ∼8 Å toward the active site channel after OAS enters the channel and reacts with active site pyridoxal 5′phosphate (PLP) (shown in yellow). With α-helix5, the N-terminal domain constituting residues from 65 to 125 moves as one unit to close the active site channel. Therefore, it is reasonable to expect that front-line residues of movable domain that line the active site entrance should be the first set of residues that come in contact with the incoming OAS. Indeed, a ternary complex crystal structure ((PDB Code: 4ORE) resolved in our recent study showed that M120 present in the N-terminus of α-helix5 is in contact with the substrate, OAS (Fig. S1*A*) (13). Therefore, we performed multiple sequence alignment and comparative structural analyses to check whether this M120 is conserved and also searched for residues that are highly conserved but undergo large structural changes when the active site of CS switches from open to closed state. Among the many residues that are highly conserved within the movable domain, we noticed a network of three conserved methionine residues, which move in tandem but exhibit large structural movements during the transition to the closed state (Fig. 1, *B*–*C*). In the open state, these three methionine residues, M120, M96, and M92, form a triangle, and they are structurally disposed at ∼20 Å from each other (Fig. 1*D*). M120 is located at the end of α-helix 5, which is connected to the α-β5 loop, a mobile loop that has no electron density in the closed state. Based on comparative sequence and structural analyses, we decided to investigate the role of this “methionine trio-network” in substrate recruitment.

### Mutation of methionine 120 significantly reduces cysteine synthesis activity

The catalytic cycle of CS comprises three distinct phases; substrate recruitment, reaction with active PLP, and product (cysteine) formation. As a first step, we examined the role of methionines in the first phase, *i.e.*, the recruitment of substrate into the active site channel. We generated 13 mutants (variants) of CS enzymes from three different bacteria (*Haemophilus influenzae*, *Salmonella typhimurium*, and *Mycobacterium tuberculosis*) as described in the methods. We targeted three methionine sites, M92, M96, and M120, and created these 13 mutants, either as point mutants or as combination mutants (double and triple mutants). For *H. influenzae* CS, we generated approximately seven mutants; *Hi*M92A, *Hi*M96A, *Hi*M120A (three single mutants), *Hi*M92 AM96A, *Hi*M92 AM120, *Hi*M96 AM120 (three double mutants, DM), and *Hi*M92 AM96 AM120A (one triple mutant, TM). For *S. typhimurium* CS, three single mutants *St*M92A, *St*M96A and *St*M120A were generated. Similarly, for *M. tuberculosis* CS, three single mutants *Mt*M92A, *Mt*M96A, and *Mt*M120A were created. All 16 proteins were purified by affinity and size-exclusion chromatography methods. Size-exclusion profiles of mutants as compared with that of their respective wild-type CS show that these mutations do not alter the oligomeric state of the protein, and all mutants elute as homodimers (Figs. S2, *A*–*C* and S3, *A*–*B*). CS and 13 mutants were further characterized by circular dichroism (CD) spectroscopy method, which indicated that secondary structural content CD signatures of mutants are similar to that of wild-type (Fig. S2*D*).

All three methionines, M92, M96, and M120, are noncatalytic residues, located 10 to 20 Å away from the reaction center. To assess their impact on cysteine synthesis activity of CS, we examined catalytic properties of 13 methionine mutants. We performed single-point activity assays at saturating substrate concentrations to examine the effect of mutations on cysteine synthesis. As shown in (Fig. S4, *A*–*C*), mutation of M120 with alanine either as single mutation or in background (*Hi*M120A, *St*M120A, *Mt*120A, *Hi*M92 AM120A, *Hi*M96 AM120A) significantly reduces the cysteine synthesis activity by ∼65 to 78%. *Hi*M96A, *St*M96A, and *Mt*M96A mutants exhibit almost similar or slightly more cysteine synthesis activity than that of WT protein, suggesting that the M96 plays very limited role in directly controlling the catalytic property of CS. Cysteine synthesis activities of M92A mutants were lower than the wild-type, but the extent of decrease was species-specific and varied from 25 to 75%. Both *St*M92A and *Hi*M92A exhibited ∼75% activity with reference to their respective wild-type enzymes, but *Mt*M92A displayed quite less, only ∼25% activity (Fig. S4*B*). In summary, mutation of M120 significantly reduced cysteine synthesis activities of CS from all three species (*Hi*CS, *St*CS, and *Mt*CS) to ∼35 to 22%.

#### Catalytic turnover rates of M120A, M92A, and M120A background mutants are significantly reduced

**Figure 2:**
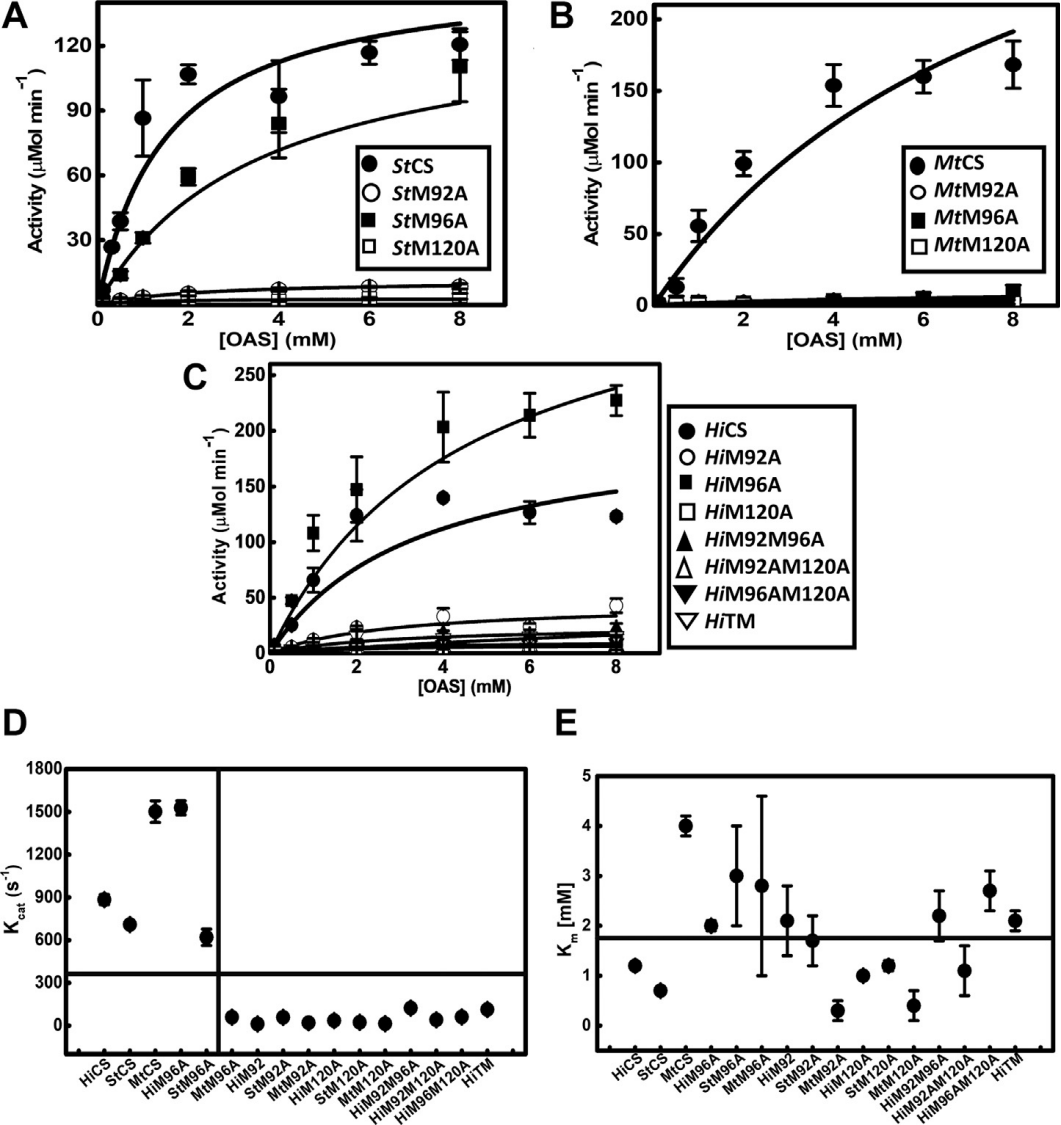
**Effect of methionine mutations on the cysteine synthesis activity of different CS mutants.** Steady-state kinetics of (*A*) *St*CS and mutants, (*B*) *Mt*CS and mutants, (*C*) *Hi*CS and mutants were performed under identical solution conditions at varied substrate concentrations. Initial velocities determined were plotted against OAS concentration. The initial velocity data, shown as mean values determined with standard error estimated at 95% confidence interval, were fit to Michaelis–Menten equation (Equation 1), and the parameters determined (K_M_, k_cat_, and k_cat_/K_M_) are provided in Table 1. *D*, parameter sensitivity plot of k_cat_*versus* enzyme type. Dichotomic behavior of k_cat_. Wild-type CS and M96A mutants except *Mt*M96A display statistically much higher k_cat_ value as compared with M120A, M92A, and M120A background mutants. *E*, Plot of K_M_*versus* enzyme type shows no trend. Horizontal and vertical lines in D and E represent the distinct pattern of distribution of k_cat_ and K_M_ parameters among various mutants.

To quantify and compare the catalytic efficiencies of mutants with that of their wild-type, we performed detailed steady-state kinetic analysis of all 16 enzymes in triplicates. As expected from single-point activity assays, amplitudes of M120A, M92A, and M120A background mutants are reduced significantly (Fig. 2, *A*–*C*). Therefore, K_M_ (apparent substrate affinity) determined from such low-amplitude kinetics data is not reliable as substrate concentration-dependent initial velocities saturate fast with very few data points in the presaturation phase. However, turnover rates (k_cat_) of these mutants can be determined more accurately as there are enough data points at the saturation phase. Kinetics of three wild-type CS and M96A mutants display high amplitude and consist of enough data points at both presaturation and saturation phases. Therefore, both K_M_ and k_cat_ for the wild-type CS and M96A mutants can be determined reliably. Initial velocities from triplicate experiments were averaged, and data were fit to Michaelis–Menten model to obtain steady-state kinetic parameters, K_M_ and k_cat_. Any parameter estimated with high uncertainty will not be sensitive to functional or structural changes. To further check the sensitivity of K_M_ to predict structural outcome, we plotted k_cat_ and K_M_ *versus* mutant/WT. The plot shows that values of K_M_ exhibit random behavior, whereas there is a clear dichotomous or categorical distribution of k_cat_ values (Fig. 2, *D*–*E*). The horizontal lines representing mean values of K_M_ and k_cat_ show that k_cat_ values of all three wild-type CS and two of M96A mutants are much higher than the average (k_cat-AV_), whereas k_cat_ values of M120A, M92A, and M120A background mutants are below the k_cat-AV_. Such categorical distribution is absent in the plot of K_M_ distribution. We tabulated the values along with fold changes for each mutant (Table 1). To estimate the (k_cat_/K_M_) for comparative analyses, we divided k_cat_ values of wild-type CS and mutants by their respective K_M_, but for M120A, M92A, and M120A background mutants, we divided their respective k_cat_ by mean of their K_M_ as the mean is better representation of K_M_ for the mutants due to high uncertainty.

**Table 1:** Steady-state kinetic parameters for CS and mutants

Protein type	K_M_ (mM) (Apparent affinity)	k_cat_ (s^−1^) (Turnover)	Fold change (WT/Mutant)	k_cat_/K_M_ (s^−1^ mM^−1^) (Catalytic efficiency)	Fold change (WT/Mutant)
*Hi*CS-WT	1.2 ± 0.03	884 ± 35 (0)	1	737 ± 34	1
*Hi*M92A	2.1 ± 0.7	12 ± 4 (0.0001)^a^	74	6 ± 2.6	123
*Hi*M96A	2 ± 0.1	1529 ± 49 (0.0001)^a^	0.6	764 ± 45	1
*Hi*M120A	1 ± 0.03	35 ± 7 (0.0001)^a^	25	35 ± 7	21
*Hi*M92 AM96A	2.2 ± 0.5	123 ± 17 (0.0001)^a^	7	56 ± 15	13
*Hi*M92 AM120A	1.1 ± 0.5	41 ± 2 (0.0001)^a^	22	37 ± 17	20
*Hi*M96 AM120A	2.7 ± 0.4	61 ± 1 (0.0001)^a^	14	23 ± 3	32
*Hi*TM^#^	2.1 ± 0.2	113 ± 2 (0.0001)^a^	8	54 ± 5	14
*St*CS-WT	0.7 ± 0.03	709 ± 25 (0.00122)^a^	1	1013 ± 56	1
*St*M92A	1.7 ± 0.5	58 ± 2 (0.0001)^a^	12	34 ± 10	30
*St*M96A	3 ± 1	620 ± 57 (0.00596)^a^	1	207 ± 71	5
*St*M120A	1.2 ± 0.1	23 ± 5 (0.0001)^a^	31	19 ± 4	53
*Mt*CS-WT	4 ± 0.2	1502 ± 76 (0.0001)^a^	1	375 ± 27	1
*Mt*M92A	0.3 ± 0.2	20 ± 0.4 (0.0001)^a^	75	67 ± 44	6
*Mt*M96A	2.8 ± 1.8	59 ± 4 (0.0001)^a^	25	21 ± 14	18
*Mt*M120A	0.4 ± 0.3	14 ± 2 (0.0001)^a^	107	35 ± 27	11

#, Triple mutant (*Hi*M92 AM96 AM120A); WT, wild type.

a (star) *p*-value (statistical significance for k_cat_) using threshold of 0.05 indicates that the difference between respective wild-type and mutants is significant.

M120A mutation has significantly reduced the turnover rate (k_cat_, 25–107 fold) and catalytic efficiencies (k_cat_/K_M-AV_, 11–53 fold) across CS. Similarly, M92A mutant also shows significantly lower catalytic efficiencies, consistent with results of single-end point assay. Turnover rates (k_cat_) of *St*M96A are very similar to that of its WT, but the (k_cat_) for *Hi*M96A is 1.6-fold more than the WT. On the contrary *Mt*M96A displays 25-fold reduced turnover (k_cat_) compared with its WT, whereas the catalytic efficiencies (k_cat_/K_M_) for *Hi*M96A are similar to that of the WT. But the catalytic efficiencies for *St*M96A and *Mt*M96A are 5- to 18-fold lesser than that of their respective WT. Results of double and triple mutants also show that M120A background mutants display significantly reduced catalytic turnover. Consistent with results of single-end point assay, mutation of noncatalytic residues M120 or M92 reduces the cysteine synthesis ability of CS significantly. Statistical analyses show that differences observed between each set of wild-types and their respective mutants are statistically significant. For example, the mean value and error determined for k_cat_ of *Hi*CS wild-type is 884 ± 35 s^−1^, which gives a range from 849 (lower bound) to 919 (upper bond). Comparison of the mean values and error range associated with k_cat_ or k_cat_/K_M_ of wild-type CS and their mutants clearly shows that the error range of mutants do not overlap with that of wild-type (Table 1). Also, we estimated *p*-value, which indicates that the observed differences between respective wildtype and mutants are statistically significant (Table 1).

#### M120A and background mutants display reduced reaction intermediate formation

Next, we examined the roles of methionine network in the second phase, PLP-reactive phase, of the catalytic cycle. Since PLP is buried at the deep end of the active site tunnel, the substrate has to travel the ∼20 Å channel to reach reaction center to enter into phase II. To dissect out which one of these three phases is most affected by methionine mutations, we examined reactabilities of mutants by monitoring the extent of reaction intermediate formation. The PLP, linked to active site lysine, is present in the form of internal aldimine and interacts with the in-coming OAS to form a number of reaction intermediates, including a more stable reaction intermediate, α-amino acrylate (28). Distinct absorption properties of α-amino acrylate and other intermediates such as geminal diamine and external aldimine (λ_max_ ∼ 340 nm, geminal diamine; λ_max_ ∼ 418 nm, external aldimine; λ_max_ ∼ 330 nm and λ_max_ ∼ 470 nm, α-amino acrylate) allow us to monitor the extent of reaction between PLP and OAS (29). The peak intensity at 470 nm should be proportional to the extent of reaction intermediate formation, and mutants with compromised substrate recruitment should show reduced signal at 470 nm.

**Figure 3:**
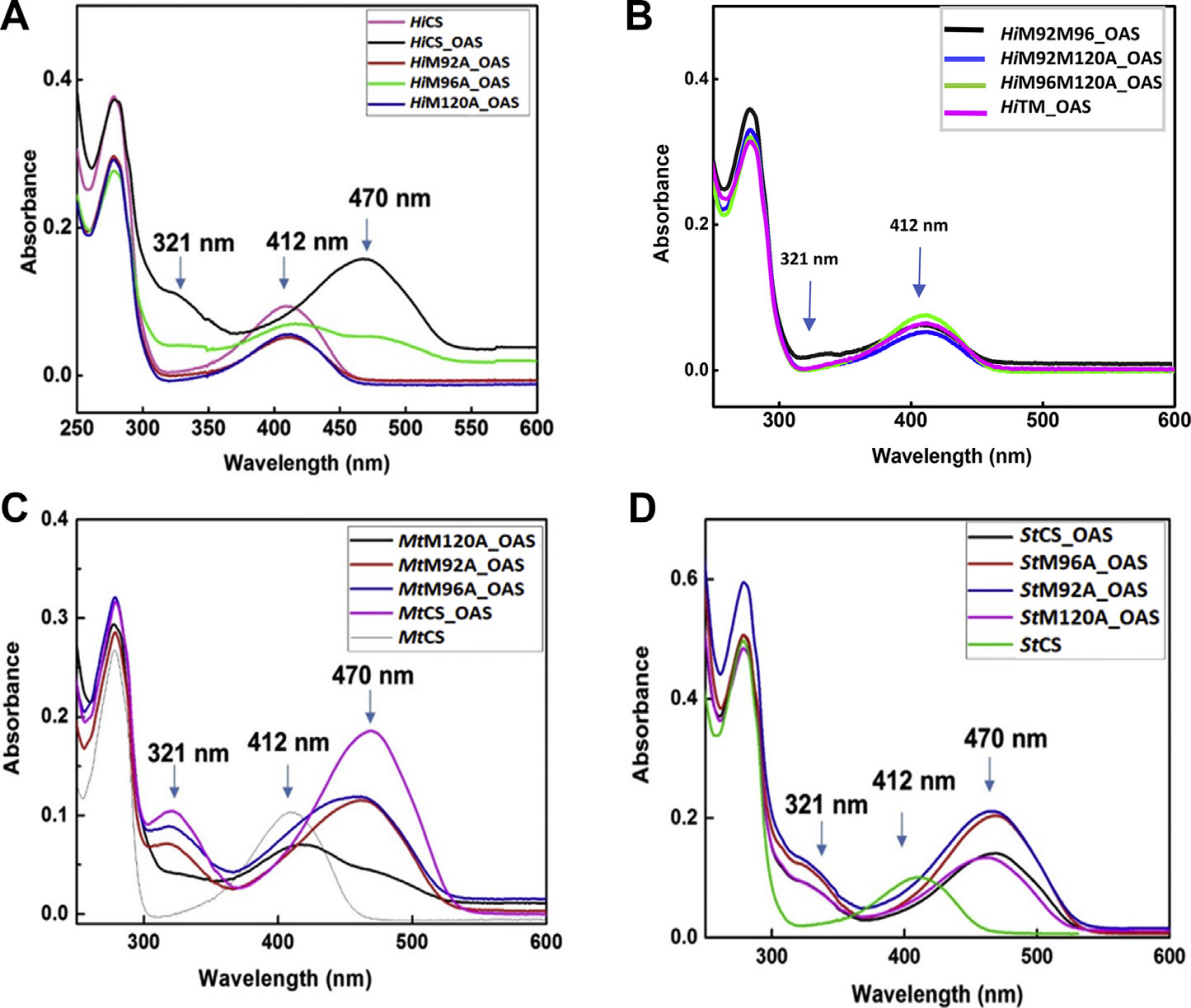
**Absorption spectroscopic analyses of α-aminoacrylate formation**. UV–visible spectra of recombinant *Hi*CS, *St*CS, *Mt*CS and in complex with the α-aminoacrylate reaction intermediate is recorded. The spectra were recorded in the buffer with 50 mMTris-Cl (pH 7.5) 100 mM NaCl and 5% glycerol. *A*–*B*, purified *Hi*CS and mutants were used to obtain the spectra of the covalent α-amino acrylate complex after the addition of 100.0 μM OAS to the enzyme solution. PLP absorbs at 412 nm and addition of ligand results in the formation of intermediates with new absorption spectra at 321 nm and 470 nm respectively. *C*–*D*, purified *Mt*CS/*St*CS and mutants were used to obtain the absorption spectra with the addition of 100.0 μM OAS. Formation of α-aminoacrylate leads to new absorbance peaks at 321 and 470 nm.

In the case of *Hi*CS, mutation of either M92 or M120 completely abrogates the formation of reaction intermediate (as both 330 nm and 470 nm peaks are not observed), but mutation of M96 has subdued effect (Fig. 3*A*). However, mutation of 96A in the M120A background (double mutant and triple mutant) of *Hi*CS displays insignificant or no reaction intermediate peaks (Fig. 3*B*). In the case of *Mt*CS, both M92A and M96A show similar but reduced intermediate formation as the magnitudes of 470 nm peaks are decreased by ∼45%. Even though *Mt*M92A mutant displays significantly reduced catalytic turnover (Table 1), the extent of formation of reaction intermediate indicated by the magnitude of 470 nm peak is higher. Mutation of either M92 or M96 affects only partially as both spectra show more or less similar signatures in *Mt*CS. The 470 nm peak of M120A of *Mt*CS is lower by 70% suggesting that mutation of M120 has more pronounced negative effect on the reaction intermediate formation (Fig. 3*C*). Similarly, mutation of M120 in *St*CS reduces the reactivity very significantly but magnitudes of 470 nm peaks of M96A or M92A are more than that of wild-type *St*CS suggesting that effect of mutations on reaction intermediate formation is not significant (Fig. 3*D*). It is interesting to note that negative effect of M96A mutation in the *Mt*CS is more pronounced, compared with that of in the *St*CS, as the relative magnitude of 470 nm peak of *Mt*M96A is reduced by ∼45%. In summary, mutation of M120 in all three species has drastically reduced the ability of CS to react with OAS, consistent with results of reduction in the cysteine synthesis activities observed above. Since reaction intermediate formation depends on the substrate flux into the channel, it is possible that mutation of M120 compromises substrate supply.

### M120 mutation affects OAS binding and recruitment

**Figure 4:**
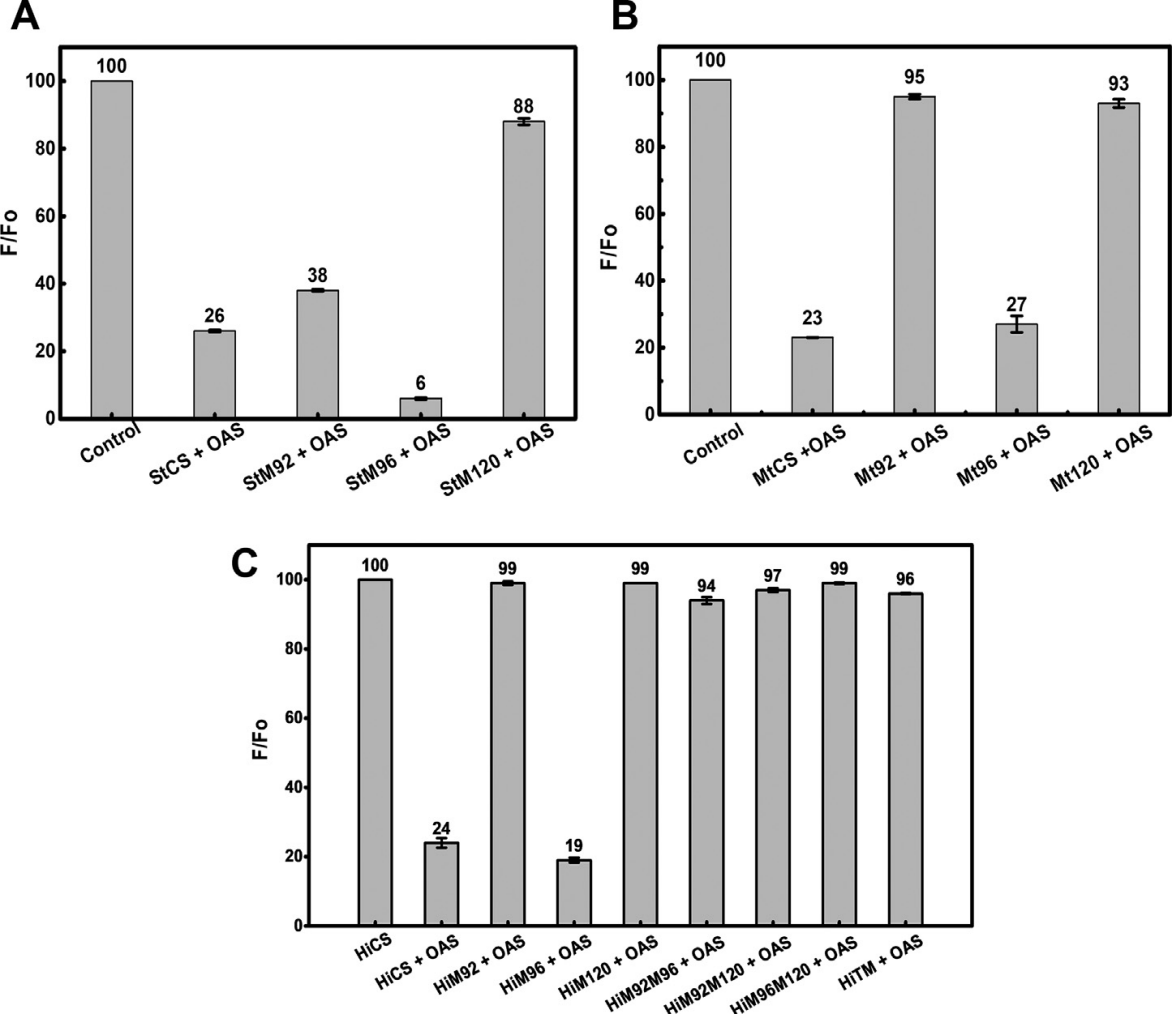
**Comparative analyses of substrate-induced fluorescence quenching (FQ) accompanied with intermediate formation and active site closing.** Protein and ligand (OAS) concentrations were kept at 0.2 μM and 2.0 μM respectively. Experimental concentrations of CS and OAS were predetermined (Fig. S5*A*). Relative normalized fluorescence change with reference to unquenched wild-type (taken as 100%, when no ligand added) is plotted against the protein type. Values on top of each bar represent the percentage of unquenched fraction (*A*) *St*CS and mutants, (*B*) *Mt*CS and mutants, (*C*) *Hi*CS and mutants. In all three cases, the relative quenching of WT is 75 to 80% and quenching of M120 mutants in the presence of OAS is 12, 7, and 3% for *St*M120A, *Mt*M120A, and *Hi*M120A respectively. The error bar represents 95% confidence interval.

Binding of OAS to all three isoforms, *Hi*CS, *St*CS, and *Mt*CS, is accompanied by change in the active site PLP fluorescence. Monitoring the extent of PLP fluorescence change at 507 nm when the protein is excited at 412 nm would allow us to quantify the extent of substrate binding and recruitment (29). As expected, PLP fluorescence at 507 nm decreases after each addition of OAS until all free enzyme molecules are saturated with OAS (Fig. 4, *A*–*C*). To find the minimum OAS concentration and equilibration time necessary to saturate fluorescence quenching at a given CS concentration (0.2 μM), we performed concentration and time range exploration experiments. Relative changes of the fluorescence quenching were scanned as a function of OAS concentrations and mixing times (Fig. S5, *A*–*B*). We determined that when ∼2.0 μM of OAS is mixed and equilibrated with 0.2 μM of CS/mutants for ∼2 min, the PLP fluorescence quenching saturates with no systematic change beyond this point. As shown in Figure 4, the fluorescence of free enzyme in the absence of OAS is set to 100%, and relative quenching is estimated by adding predetermined levels of OAS (2.0 μM) and recording the fluorescence after ∼2 min.

The relative quenching ratio (F_obs_/F_0_) is calculated as the ratio of absolute fluorescence intensity of wild-type/mutant saturated with OAS (F_obs_), normalized to the fluorescence of free wild-type/mutant CS (F_0_) at the same concentration. The percentage of relative quenching can be determined from the percentage of unquenched fraction reported on top of each bar (Fig. 4, *A*–*C*). While three wild-type CS display quenching in the range of ∼75 to 77% (23–26% unquenched), M120A mutants show very low quenching in the range of ∼3 to 12% (>88% unquenched). The ∼75% quenching achieved for wild-type under fixed experimental conditions suggests that the ratio of enzyme–substrate complex to free enzyme is similar for three wild-type CS. Similar to wild-type CS, M96A mutants also show high percentage of quenching in the range of 73 to 94%. However, OAS binding-induced fluorescence quenching of M92A mutants shows species dependency with *St*M92A showing 62% quenching as compared with 1 to 5% quenching of *Hi*M92A and *Mt*M92A mutants. The percentage of quenching, F_obs_/F_0_, is proportional to the fraction of enzyme·substrate (CS·OAS) complex, and higher quenching percentage corresponds to better ability of that mutant to recruit OAS and form enzyme·substrate (CS·OAS) complex. Consistent with results of single-point activity and steady-state kinetic studies, M120A mutants across the species showed very low quenching (12% or less, ∼90% unquenched) as compared with that of their respective wild-types and other mutants. Similarly, double and triple mutants of *Hi*CS (M92 AM120A, M96 AM120A, and M92 AM96 AM120A) with M120A mutation also showed significantly reduced quenching (Fig. 4*C*). Both M96A and M92A mutants display species-dependent quenching, but M96A behaves oppositely to M92A. M96A fluorescence quenching is very sensitive to OAS like that of wild-type, whereas the quenching of M92A is insensitive to OAS, like that of M120A mutant. Together, fluorescence quenching results suggest that mutation of M120A almost abolishes the OAS binding (>90%) and M92A may also plays a role in OAS binding although to a lesser extent.

### Pre-steady-state kinetics of OAS binding

**Figure 5:**
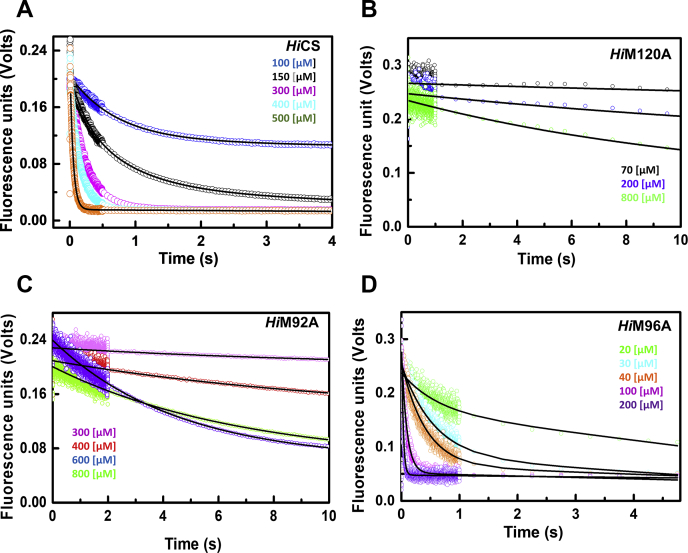
**Comparative pre-steady-state kinetic fluorescence analyses of OAS binding (*Hi*CS and mutants).** Active site-bound PLP of proteins is excited at 412 nm and fluorescence emission is recorded using 455 nm long-pass filters. Concentrations of OAS are shown within each plot. *A*–*D*, kinetics of fluorescence quenching at different OAS concentrations; *A*) *Hi*CS wild-type, *B*–*D*) Mutants—*Hi*M120A, *Hi*M92A, and *Hi*M96A. OAS binding kinetics of M120A and M92A are much slower as compared to that of wild-type *Hi*CS and *Hi*M96A at a given OAS concentration (Compare traces from panels *A* and *D* with traces from *B* and *C*). All traces were fit to single exponential model and rates and associated errors estimated with 95% confidence intervals are given in Table 2. Complete traces of *Hi*M120A and *Hi*M92A are shown in (Fig. S7) for clarity.

**Figure 6:**
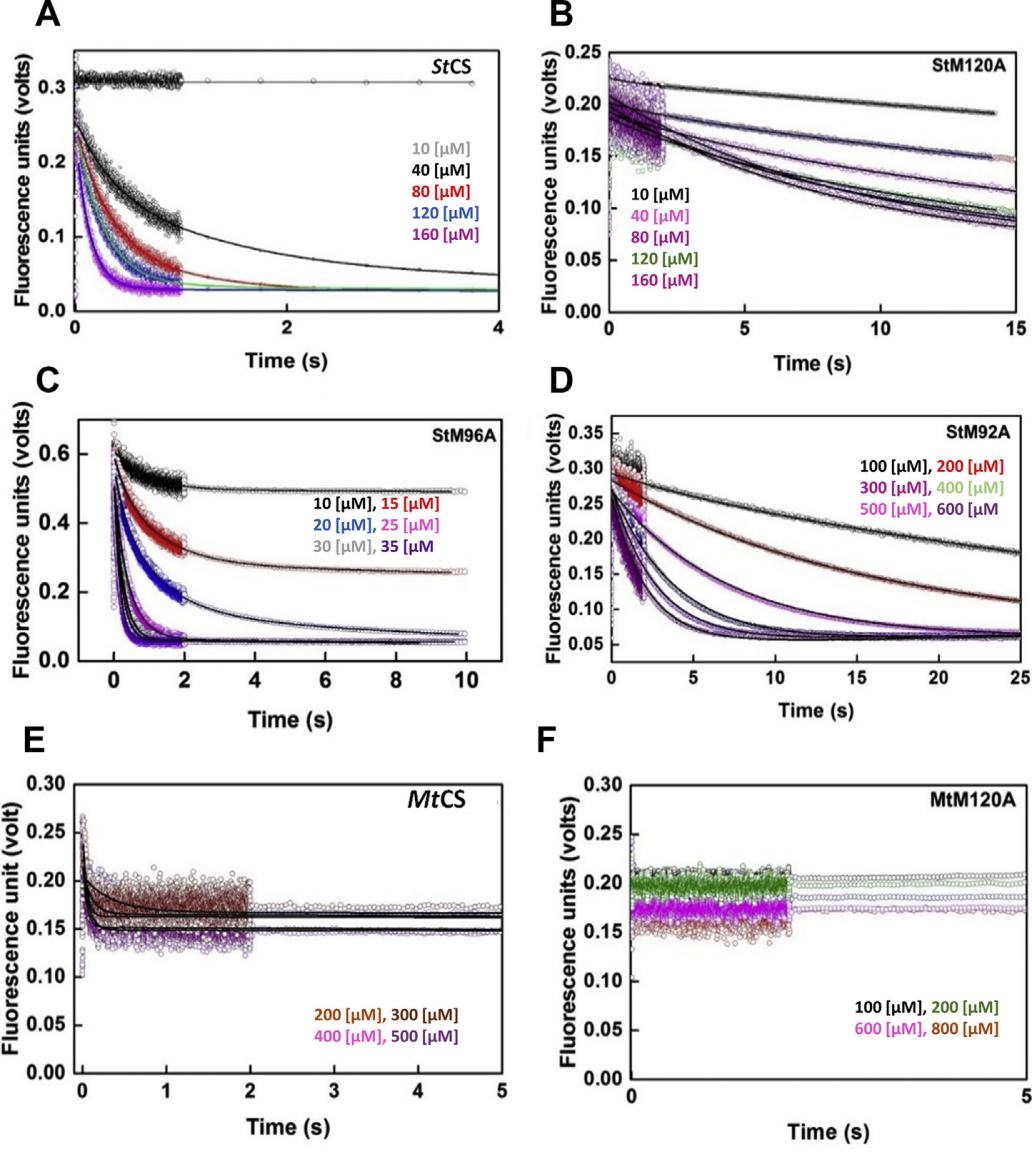
**Comparative pre-steady-state kinetic fluorescence analyses of OAS binding (*St*CS, *Mt*CS, and mutants).** Active site-bound PLP of proteins is excited at 412 nm and fluorescence emission is recorded using 455 nm long-pass filters. Concentrations of OAS are shown within each plots. *A*–*D*, kinetics of fluorescence quenching at different OAS concentrations; *A*) *St*CS wild-type, *B*) *St*M120A, *C*) *St*M96A, *D*) *St*M92A. Substrate-binding kinetics of M120A is much slower as compared with wild-type. *E*, *Mt*CS wild-type, *F*, *Mt*CSM120A. Fluorescence signal of *Mt*M120A mutant did not change upon mixing with substrate and the data could not be fit. Much slower rates of OAS binding kinetics of M120A and M92A as compared with that of wild-type enzymes at a given OAS concentration (Compare kinetic traces of wild-type with that of mutants). All traces were fit to single exponential model and rates and error estimated with 95% confidence interval are given in Table 2.

Results of equilibrium experiments clearly show that M120 is directly involved in OAS binding and recruitment. In order to understand the mechanism by which M120 facilitates OAS recruitment, we used pre-steady-state approaches for monitoring the kinetics of OAS binding (Figs. 5 and 6). Similar to equilibrium approach, a decrease in the intrinsic PLP fluorescence upon OAS addition was continuously monitored for determining the rates of OAS binding to different mutants. Rates measured as a function of OAS concentrations under similar solution conditions were used for estimating on-rate (k_on_) constants (Table 2). All experiments were performed under pseudo-first-order conditions (excess of OAS). All pre-steady-state kinetic time traces showed single exponential decay that approaches same plateau value corresponding to approximately >95% of quenching of initial PLP fluorescence. The extent of quenching suggests that the kinetic time course reflects the formation of closed-state CS·reaction-intermediate complex. As expected, rates of PLP fluorescence quenching signal increase with increasing OAS concentration. Traces in Figure 5 were fit to single exponential decay model using Equation 6 to obtain values of rates (k_obs_) at each OAS concentration. Kinetic time traces and fits to traces for wild-type *Hi*CS and three methionine mutants, *Hi*M120A, *Hi*M92A, and *Hi*M96A, are shown (Fig. 5, *A*–*D*). Rates of OAS binding to *Hi*M120A and *Hi*M92A are much lower than that of wild-type at any given concentration of OAS.

**Table 2:** Summary of pre-steady-state kinetics data for the OAS binding to the CS

Enzyme	Rapid kinetics (association rate constant) k_on_ (M^−1^ s^−1^)	Fold change (WT/Mutant)
*Hi*CS	(4.0 ± 0.08) × 10^4^	1
*Hi*M120A	(1.4 ± 0.08) × 10^2^	286
*Hi*M96A	(1.4 ± 0.08) × 10^5^	0.3
*Hi*M92A	(5 ± 0.08) × 10^2^	80
*Hi*M120 AM92A	(2.0 ± 0.08) × 10^2^	200
*Hi*M92 AM96A	(1.0 ± 0.08) × 10^3^	40
*Hi*M92 AM96 AM120A	(1.9 ± 0.08) × 10^3^	21
*St*CS	(2.1 ± 0.08) × 10^4^	1
*St*M120A	(1.0 ± 0.08) × 10^3^	21
*St*M92A	(6.8 ± 0.17) × 10^2^	31
*St*M96A	(8.8 ± 0.5) × 10^4^	0.2
*Mt*CS	(2.9 ± 0.05) × 10^4^	1
*Mt*M120A	NA	
*Mt*M92A	NP	
*Mt*M96A	NP	

NA, data couldn’t be fitted; NP, could not be performed.

**Figure 7:**
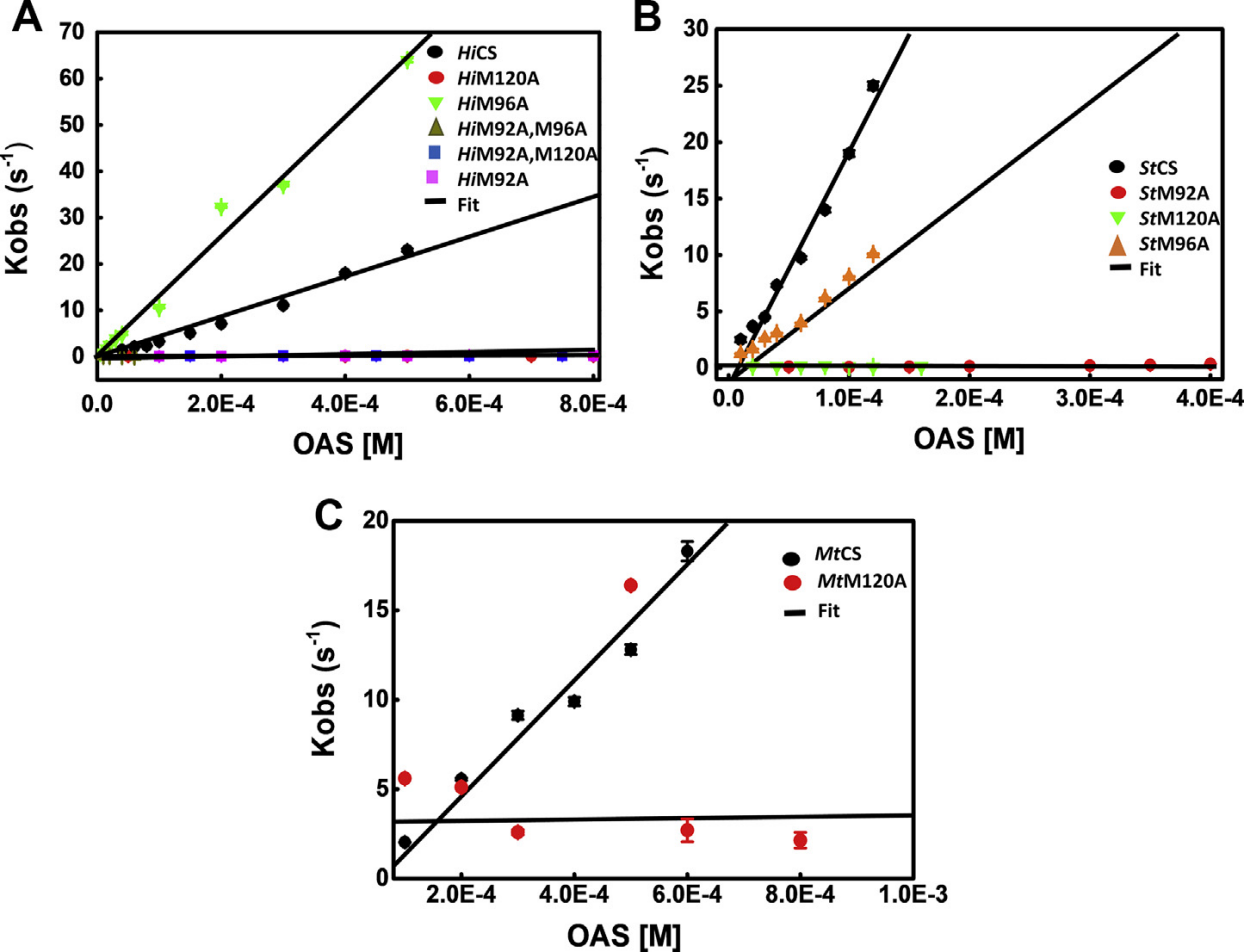
**The plots of observed rates of OAS binding as a function of OAS concentration.** The observed rates (k_obs_), determined by fitting the fluorescence quenching traces to single exponential model, are plotted against OAS concentration (Table 2). Each data point represents observed rates determined from experiments and associated errors were estimated with 95% confidence interval. The representative lines were drawn to show the trend of data, not lines from fitting to any model. *A*, plot of K_obs_*versus* [OAS] for *Hi*CS and mutants. *B*, plot of K_obs_*versus* [OAS] for *St*CS and mutants. *C*, Plot of K_obs_*versus* [OAS] for *Mt*CS and mutants. The observed rates of M120A and M92A as compared with that of wild-type enzymes show that these mutants are defective in binding to OAS.

Similarly, kinetics of OAS binding by *St*CS, *Mt*CS, and methionine mutants of these two proteins were also performed under same solution conditions. Observed rates estimated from single exponential fit were plotted as a function of OAS concentration. As shown in (Fig. 6), under pseudo-first-order conditions ([OAS] >> [CS]), the observed rates (k_obs_) increase linearly with increasing substrate (OAS) concentration. The linear least squares fit to the rates plotted *versus* OAS concentrations yields the slope (on-rate constant, k_on_) and the intercept, (off-rate constant, k_off_) (Equation 6) (Fig. 7). On-rate binding constants of all three wild-type enzymes and that of other ten mutants are shown (Table 2). All three wild-type CS show similar rate of substrate binding with one- to twofold changes as compared with low on-rates of substrate binding displayed by M120A and M92A mutants of *Hi*CS and *St*CS. Fast kinetics data for *Mt*CS120A could not be fitted as there was no signal change when mixed with OAS. Pre-steady-state data for Mt96A and Mt92A could not be performed due to their high propensity to aggregate at higher concentrations. As seen in the gel filtration purification profile (Fig. S2*C*), the *Mt*CS and its mutants were purified at lower concentrations. Both mutants aggregate upon concentrating above 15.0 μM.

Upon comparison of reduced on-rates of two M120 mutants, the *St*M120A binds approximately seven- to eightfold faster rate than the *Hi*M120A, suggesting a species-specific effect of mutation. As shown in Table 2, on-rate binding constants of mutants of *Hi*M120A, *Hi*M92A, and *Hi*M120 AM92A are ∼286-fold, 80-fold, and 200-fold lower than that of *Hi*CS wild-type. The estimated error ranges (∼95% confidence interval) of respective WT CS and mutants do not overlap, suggesting that the observed differences are statistically significant. As expected, on-rates of both *Hi*M96A and *St*M96A are three- and fivefold higher than that of their respective wild-type CS. *St*M120A and *St*M92A mutants show 21- to 31-fold reduction in the substrate recruitment rate. These results clearly demonstrate that the very first step in substrate recruitment is controlled by these noncatalytic surface residues, M120 and M92. Mutation of M120 reduces the recruitment rate by ∼20- to 286-fold, severely affecting the first step of contact between OAS and CS. The negative intercepts of wild-types indicate that k_on[OAS]_ >> k_off_ (Fig. 7). This is real because, the OAS is trapped as α-aminoacrylate within the active site of CS and its dissociation from the active site is very slow. Therefore, M120 plays a crucial role in the very first step of substrate binding.

### Role of methionine-trio in inhibitor binding

The active site of CS is also the binding site for C-terminal of SAT, which is the natural binding partner as well as inhibitor of CS. Both equilibrium and knietic approaches provided direct evidence for the role of M120 in recruiting the substrate, and this result also validates our previous structural observation (PDB code: 4ORE) in which M120 makes the contact with the substrate. In two ternary complexes resolved in that study (PDB code: 4ORE and PDB code: 4ZU6), M120–substrate contact at the active site entrance has resulted in subtle but very conspicious conformational changes deep within the active site, ∼17 to 20 Å away from the site of M120 contact with the substrate. For example, curical hydrogen bonds between the last C-terminal ILE residue of the inhibitor and peptide binding loop of CS are broken, weakening the interaction between the enzyme and inhibitor (13). Therefore, we examined the contribution of M120 to the inhibitor affinity even though, M120 does not interact with inhibitor peptide in crystal structure resolved. Since the contact between M120 and substrate disengages the inhibitor, the contribution of M120 in favor of substrate can be estimated from the contribution of M120 to inhibitor binding in the absence of substrate.

**Figure 8:**
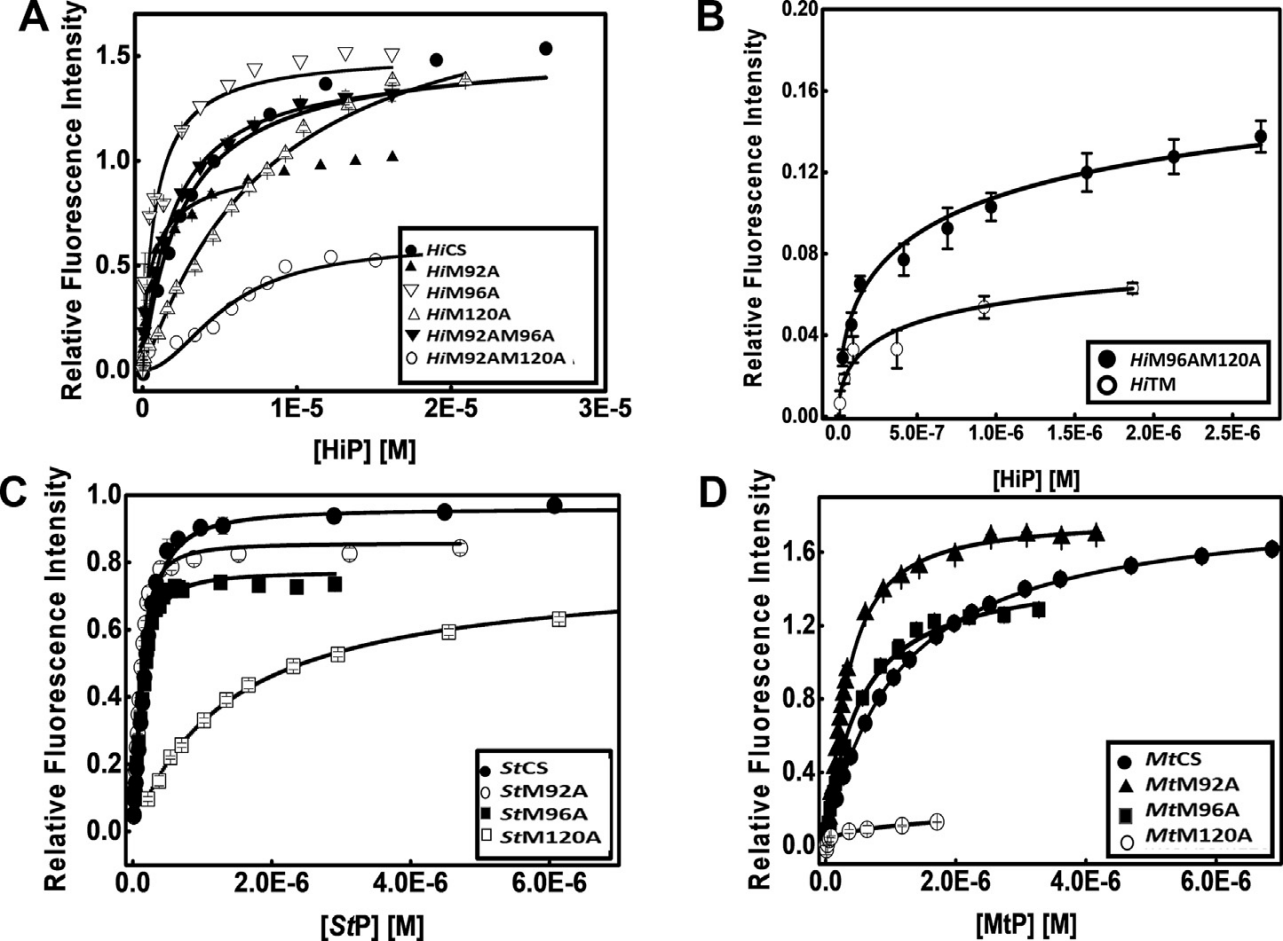
**Equilibrium binding studies of SAT C-terminal peptides/C10 peptides (inhibitor) binding to CS and mutant enzymes.** Relative fluorescence increase at 507 nm upon C10 peptide binding was monitored and binding constants were determined. Binding of C10 peptides to *Hi*CS and its mutants (*A*–*B*) *St*CS and its mutants (*C*), *Mt*CS and its mutants (*D*). Determined dissociated constants are given in Table 3. The M120A has showed decreased affinity for C10 peptide, but M92A and M96A mutants display increased affinities.

Similar to previous studies, we used ten residue SAT C-terminal peptides as the high-affinity inhibitor and kept the enzyme concentration in the range of 0.2 to 0.4 μM (30). The extent of complex formation was quantified from the changes in PLP fluorescence at 507 nm (Fig. 8). Binding isotherms were analyzed using two similar binding sites model and equilibrium dissociation constants K_d_ (μM) were determined (Table 3). M120A mutation reduced the affinity for inhibitor peptides by 3, 5, and 1.4-fold in *Hi*CS, *St*CS, and *Mt*CS, although the reduction in *Mt*CS was statistically insignificant. On the contrary, both M92A and M96A background mutations seem to favor the substrate-antagonistic SAT C-terminal peptide binding, although in a species-dependent manner. In *Hi*CS, mutation of M96 increases the affinity by approximately fivefold (3.4 μM *versus* 0.65 μM), but the affinity increases by 24- to 34-fold in the double and triple mutant backgrounds (*Hi*M96 AM120A and *Hi*M92 AM96 AM120A). As shown in Table 3, only M96A mutation is able to reverse the negative effect of M120A mutation (please compare the affinities of *Hi*M92 AM120A *versus Hi*M96 AM120A). *Hi*M92A exhibits approximately fivefold increase but in the M120A background, M92A mutation fails to reverse the effect of M120A. In *St*CS, *St*M92A and *St*M96A increase the inhibitor binding affinity by 1.5- to 3.0-fold, and *Mt*M92A and *Mt*M96A mutants show two- to fourfold higher affinity toward peptide as compared with the wild-type *Mt*CS. In summary, the effect of M120 mutation on inhibitor peptide binding is not as profound as compared with substrate binding. Nevertheless, M120 contributes to SAT C-terminal peptide binding, by three- to fivefold in *Hi*CS and *St*CS.

**Table 3:** The equilibrium binding constants K_d_ of peptide binding for the wild-type CS and mutants

Protein type	Equilibrium binding dissociation constant (K_d_) (μM)	Fold change (WT/Mutant)
*Hi*CS	3.4 ± 0.11	1
*Hi*M120A	10 ± 0.8	0.3
*Hi*M96A	0.65 ± 0.07	5
*Hi*M92A	0.72 ± 0.15	5
*Hi*M92 AM96A	0.63 ± 0.13	5
*Hi*M92 AM120A	9.2 ± 3	0.4
*Hi*M96 AM120A	0.14 ± 0.03	24
*Hi*M92 AM96 AM120A	0.1 ± 0.02	34
*St*CS	0.29 ± 0.02	1
*St*M120A	1.5 ± 0.04	0.2
*St*M92A	0.11 ± 0.01	3
*St*M96A	0.19 ± 0.02	1.5
*Mt*CS	1.0 ± 0.01	1
*Mt*M120A	1.37 ± 1.6	0.7
*Mt*M92A	0.28 ± 0.06	4
*Mt*M96A	0.5 ± 0.05	2

Error range associated with the K_d_ value of *Mt*M120A is large and overlaps with confidence interval range estimated for *Mt*CS.

### Structural analyses of mutants reveal two different inhibitor binding conformations

To understand how M120 and other two methionine residues influence substrate selection at molecular level, we crystalized and resolved high-resolution structures of four mutants (M92A, M96A, M120A, M120 AM92A) (Table 4). Results show that point mutations do not alter the overall structure of the enzyme, but notable conformational changes observed at the mutated site as well as within the reaction center allow us to explain the effect of mutations on affinities of substrate and inhibitor. In four crystal structures resolved in this study, “TSGNT loop” assumes “pre-inhibitor” binding pose in M92A and M96A structures (PDB codes: 7CM8 and 7C35) and in the M120A and M92 AM120A structures (PDB codes: 5XCN and 5XCW), “TSGNT loop” assumes “post-inhibitor” binding pose. The loop moves in opposite directions (∼5.3 Å between pre- and postbinding conformations) with reference to the position of loop observed in the wild-type *Hi*CS structure. Opposite movement of loop directly correlates with the opposite trends of inhibitor binding affinities of M120 mutants *versus* M92 and M96 mutants, with prebinding poses favoring CS·inhibitor stability and postbinding pose opposing the peptide binding.

**Table 4:** Crystallographic data collection and refinement statistics

*Hi*CS (PDB ID)	M92A (7CM8)∗	M96A (7C35)	M120A (5XCN)	M92 AM120A (5XCW)
Data collection				
Wavelength	1.541 Å	1.541 Å	1.541 Å	1.541 Å
Space group	I 41	I 41	I 41	I 41
Unit cell (A, B, C,) Å	112.44 112.44 44.00	113.01 113.01 44.05	112.27 112.27 46.09	112.55 112.55 46.35
Resolution range	35.56 Å–1.9 Å (1.96–1.9) Å	35.74 Å–2.10 Å (2.17–2.1) Å	28.07 Å–1.69 Å (1.75–1.69) Å	34.09 Å–1.89 Å (1.95–1.89) Å
R_merge_	0.15 (0.82)	0.04 (0.22)	0.05 (0.32)	0.07 (0.39)
CC_1/2_	0.99 (0.68)	0.99 (0.86)	0.99 (0.99)	0.99 (0.85)
Overall I_Avg_/sigma_Avg_(I)	9.6 (2.7)	10.1 (3.2)	28.9 (2.2)	28.3 (3.7)
Completeness (%)	98.9 (98.6)	99.5 (1.00)	94 (0.88)	100 (0.93)
Total reflections	221,588	32,004	30,253	23,458
Unique reflections	21,690 (1380)	16,477 (1643)	30,261 (2821)	23,438 (2301)
Refinement				
Resolution range	35.56 Å–1.9 Å	35.74 Å–2.10 Å	28.07 Å–1.69 Å	34.09 Å–1.89 Å
R-work	0.19 (0.37)	0.20 (0.23)	0.16 (0.27)	0.16 (0.23)
R-free	0.21 (0.42)	0.22 (0.34)	0.19 (0.31)	0.20 (0.30)
Number of nonhydrogen atoms	2346	2261	2551	2474
Macromolecules	2258	2261	2316	2291
Protein residues	309	302	313	310
Average B-factor	32.03	38.17	26.22	23.43
Macromolecules	32.01	38.07	25.41	22.74
Solvent	32.52	39.28	34.18	32.07
RMS (bonds)	0.018	0.015	0.007	0.007
RMS (angles)	2.11	1.87	1.11	1.08
Ramachandran favored (%)	93.38	95.09	97	97
Ramachandran allowed (%)	6.29	4.56	2.5	2.9
Ramachandran outliers (%)	0.33	0.35	0	0
Rotamer outliers (%)	1.75	1.93	0.84	0.85
Clashscore	2.22	2.35	0.64	0.86

Statistics for the highest-resolution shell are shown in parentheses.

I/Avg sigma (I): ratio of average intensity and average uncertainty.

I/Avg sigma (I): overall average (I/sigma (I)) of the data set.

**Figure 9:**
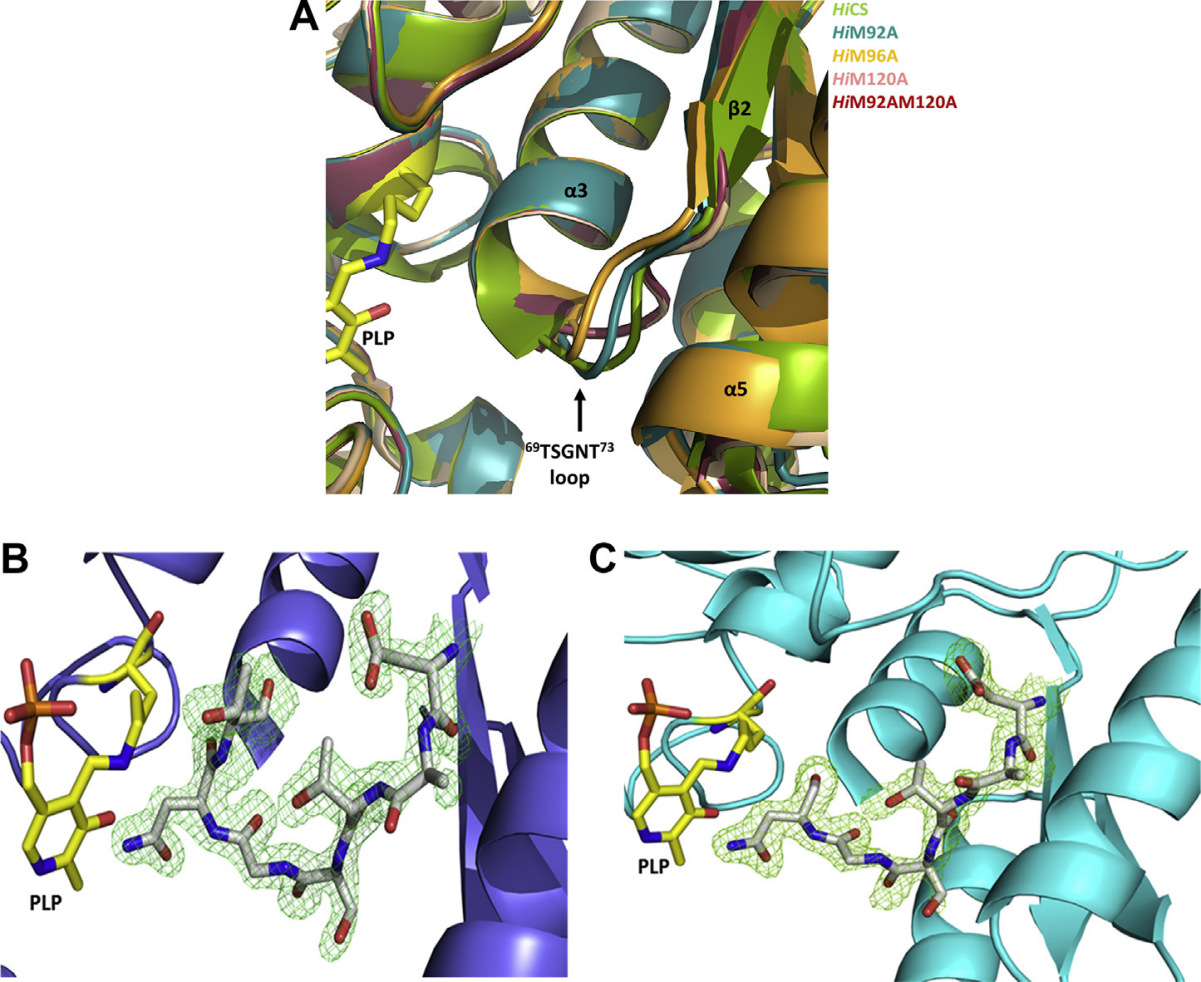
**Structural analyses of active site conformation of methionine mutants.** High-resolution crystal structures of M92A, M96A, M120A, and M120 AM92A mutants of *Hi*CS were resolved (Table 4). *A*, superposition of *Hi*CS and its mutant structures shows different conformations of active site “TSGNT” loop (which binds both substrate and inhibitor (SAT C-terminal). While the “TSGNT” loop of M120A superposes very well with that of M92 AM120A but conformations of “TSGNT” loop of M92A and M96A are different, they move in opposite directions. The polarized movement of this loop may explain the decreased affinity of M120A *versus* increased affinities of M92 and M96 mutants toward SAT C10 peptide binding. *B*–*C*, simulated annealing *Fo-Fc* omit maps of “TSGNT” loop of M120A (*B*) and M92 AM120A double mutant (*C*), contoured at 2.8σ.

Conformations of both α5-β4 loop of active site entrance and substrate/inhibitor binding “TSGNT” loop are altered in the M120A and M92 AM120A structures. In the wild-type CS, side chain of M120 interacts with the main-chain amino group of S70 and main-chain carboxyl group of A69 of “TSGNT” loop at 3.8 Å and 3.7 Å, respectively (Fig. S6, *A*–*B*). Similarly, M92 interacts with main-chain carboxylic group of T69 with the distance of 3.5 Å and M96 interacts with main-chain carboxylic group of S70 at a distance of 3.7 Å. All these interactions hold the “TSGNT” loop in the “reference” conformation, that is in between “pre and post” binding conformations (Fig. 9, *A*–*C*). Mutation of M120 disrupts the interaction between S70 and A68 of the peptide loop and pushing the loop ∼3.8 Å further away to postbinding conformation. This reduces the chances of incoming inhibitor to latch onto the binding loop as compared with reference conformation present in the wild-type, thus reducing the affinity of inhibitor. In the case of M96 mutation, the interaction between M92 and the carboxylic group of T69 is broken, and the loop moves in the opposite prebinding conformation, more close toward the active site channel. This move increases the chances of inhibitor contacts with the binding loop, therefore leading to increased binding affinities of inhibitor for M96 and M92 mutants. Our study presents the first structural evidence to show that the substrate/inhibitor binding loop may adopt two different conformations.

## Discussion

We employed an integrated approach to provide a detailed view of how CS is able to selectively recruit its substrate, OAS, in the presence of a high-affinity natural inhibitor, SAT. SAT and CS catalyze two consecutive steps of cysteine biosynthesis pathway in plants and bacteria (14, 16). Both associate to form a highly stable CRC complex in which the cysteine synthesis activity of CS is significantly reduced (17). Therefore, it remained elusive until we showed recently that CS employs a novel “competitive-allosteric” mechanism to recruit its substrate even when the active site of CS is bound with SAT C-terminal, referred as inhibitor of CS (13). This study was undertaken to trace and dissect out molecular features that allow CS to recognize and recruit its substrate in the presence of natural inhibitor. To generalize our findings, CS from three different species and ∼13 mutants of these three different versions were analyzed and compared for cysteine synthesis activity, substrate recruitment, inhibitor binding by using a combination of high-resolution approaches.

Single-point enzyme activity, steady-state kinetics, fluorescence quenching, and pre-steady-state kinetic studies clearly show that mutation of M120 reduces the cysteine synthesis activity by significantly compromising substrate recruitment abilities of all three wild-type CS. We employed rapid kinetic binding approaches for comparing forward rate constants of binding with catalytic turnovers determined for mutants. The high-resolution approach allowed us to map the role of M120 to the first phase of substrate recruitment of CS. Results are consistent with earlier structural observation that M120 makes the first contact with the incoming substrate (1). Therefore, this study establishes the role of M120 in selective substrate recruitment unambiguously. M120 contributes to selective substrate recruitment by enhancing affinity for substrate and selectively dissociating the bound inhibitor. Our extensive structural analyses showed that the crucial hydrogen bonds between the active site residues and inhibitor peptide are broken when M120 makes the first contact with the incoming substrate (13). In this study, we quantified the contribution of M120 in favor of substrate by measuring the changes in the on-rate of substrate binding and inhibitor binding. Therefore, the net discriminative effect in favor of substrate can be estimated by combining the contribution from both. In *Hi*CS, M120 favors substrate binding by a factor of ∼858-fold as M120A mutation reduces the rate of substrate binding by 286-fold and peptide binding by threefold. In *St*CS, the net discriminative force estimated from multiplying on-rate of substrate binding/catalytic turnover and peptide dissociation is ∼100 to 150 fold. Together, our study unravels that M120 acts as a selectivity filter against the inhibitor with net discriminative force of 100- to 858-fold in favor of substrate. Since the first step of substrate recruitment is affected, substrate flux into the ∼20 Å channel is significantly reduced, resulting in manifold reduction in reaction rate and product formation. Mutation of M92 also affected the substrate recruitment although to a lower extent. M120 emerges as the primary and the most important residue in substrate recruitment by CS.

High-resolution crystal structures of four mutants provide insights into how M120 modulates inhibitor affinity by “engagement and disengagement” mechanism in the absence and presence of substrate. In the absence of substrate, M120 interaction with inhibitor/substrate binding “TSGNT” loop stabilizes the CS·Inhibitor complex, but M120 disengages the “TSGNT” loop as soon as it senses the substrate at the active site entrance. The “engagement and disengagement” switch is a gate-like allosteric mechanism that empowers M120 to facilitate the dissociation of inhibitor from the active site when it engages with substrate at the entrance. The equilibrium constants of inhibitor binding to M120 and other mutants allowed us to estimate the contribution of M120 to inhibitor in the absence of substrate. The three- to fivefold reduction in the affinity of inhibitor is an indirect measure of contribution of M120 to substrate recruitment when the substrate makes contacts with M120, but if we compare the contribution of all M120 and M92. Results presented here describe the first systematic study to explore the features of substrate recruitment of CS in the presence of natural high-affinity inhibitor, SAT. Results obtained from multiple independent and orthogonal approaches map the role of noncatalytic residue M120 in facilitating the substrate recruitment. Significantly reduced rate of substrate binding may suggest that substrate binding has become the rate-limiting step in the M120A mutants. Formation of α-aminoacrylate is characterized as the reaction intermediate in CS. However, to conclude substrate binding as the rate-limiting step, rate of reaction of substrate with PLP, rate of conformational transition to closed state, rate of intermediate formation, and rate of product release of all mutants need to be determined and compared with rates of substrate binding determined in this study. Together, our results estimate that CS use M120 to discriminate in favor of substrate at least by ∼100- to 858-fold by employing the allosteric engage (substrate) and disengage (inhibitor) mechanism.

A detailed understanding of both catalytic and substrate selection mechanisms has many applications, ranging from tuning the substrate selectivity of enzymes toward the desired “substrate-product conversion” to identify critical steps in the whole catalytic cycle to design target-specific therapeutic molecules. Our data provides a framework for identifying key features of substrate selectivity, which enable CS to discriminate the high-affinity natural inhibitor protein against its substrate. CS and SAT associate to form a high-affinity CRC complex (15, 16, 17, 31). Multiprotein/enzyme complexes are increasingly being targeted for developing small molecule–based therapeutic intervention for a variety of diseases (32, 33, 34, 35). However, selective targeting remains a major challenge to drug discovery groups due to the paucity of information about details of complex formation and dissociation. Enzymes of cysteine biosynthesis pathway, including CS (CysK), have been considered as potential drug targets (36, 37, 38, 39, 40). Using structure-based *in silico* screening, multiple studies have identified several inhibitors against CS and its homologue, CysM (41). Results presented open prospects for designing selective inhibitors that bind through competitive-allosteric mechanism by exploiting M120-based selective recruitment mechanism.

## Methods

### Bioinformatics analysis

A total number of 18-homologous protein sequences of CS were taken for the multiple sequence alignment from a wide range of bacteria and plants from the NCBI database. Redundancy was eliminated by taking only one protein sequence from one genus. The alignment was performed using CLUSTAL-W (42), freely available at EBI website, and online multiple sequence alignment tool Multalin (43).

### Site-directed mutagenesis

Mutations at M120, M92, and M96 were introduced into the wild-type sequences of CS from three different microorganisms (*M. tuberculosis*, *Haemophilus influenzea*, *S. typhimurium*) using quick-change site-directed mutagenesis protocol (Agilent technologies, Inc). The primers containing the mutations were synthesized by IDT, Inc, USA. Mutations in the CS genes were confirmed by DNA sequencing. We generated 13 mutants (variants) of CS enzymes from three different bacteria (*H. influenzae*, *S. typhimurium*, and *M. tuberculosis*). We created 13 mutants, either as point mutants or as combination mutants (double and triple mutants). For *H. influenzae* CS, we generated approximately seven mutants; *Hi*M92A, *Hi*M96A, *Hi*M120A (three single mutants), *Hi*M92 AM96A, *Hi*M92 AM120, *Hi*M96 AM120 (three double mutants, DM), and *Hi*M92 AM96 AM120A (one triple mutant, TM). For *S. typhimurium* CS, three single mutants *St*M92A, *St*M96A, and *St*M120A were generated. Similarly, for *M. tuberculosis* CS, three single mutants *Mt*M92A, *Mt*M96A, and *Mt*M120A were created.

### Protein expression and purification

Coding frames of CS, from *S. typhimurium* (*StCS*) strain LT2, *H. influenzae (HiCS)*, and *M. tuberculosis* (*MtCS*), were cloned into N-terminal 6His-pET28a+ expression vector. Similarly, wild-type and mutant constructs were expressed in *Escherichia coli* BL21DE3 cells and purified. The N-terminal His-tag of all enzymes was removed by thrombin digestion and further purified by size-exclusion chromatography. Purified fractions were analyzed on a 12% SDS-PAGE gel and found to be >95% pure. We determined protein concentrations using molar extinction coefficients of *Hi*CS, *St*CS, *Mt*CS (21,555 M^−1^ cm^−1^, 19,940 M^−1^ cm^−1^, 11,500 M^−1^ cm^−1^ respectively) estimated at 280 nm. The enzyme assay for three wild-types and all mutants was carried out using the acid ninhydrin assay for cysteine formation as described (44).

### Secondary structure analyses by circular dichroism

CD measurements were carried out with a JASCO spectropolarimeter (Jasco, Tokyo, Japan) equipped with a Peltier-type temperature controller (PTC-348W). Far-UV spectra were obtained in a quartz cuvette with a 10 mm light path length, and each spectrum obtained was an average of seven scans. The ellipticities of protein CD spectra are subtracted from reference buffer spectrum and reported as mean residue ellipticity (MRE) in degcm^2^/dmol units.

### Single-point activity and steady-state kinetics of enzymes

The single-point activity and detailed steady-state kinetics assays were carried out to assess the effect of mutations. Both assays were performed using the acid ninhydrin method for quantifying the amount of cysteine (44). The substrate, O-acetylserine (OAS, manufactured by Sigma) was dissolved in 0.1 M HEPES pH 7.0. CS hydrolyzes OAS in the presence of Na_2_S and synthesizes cysteine. Formation of cysteine is monitored at 560 nm (extinction coefficient of cysteine is 28,000 M^−1^ cm^−1^). Both single-point activity and steady-state kinetics assays were performed at 30 °C in 0.1 M HEPES, at pH 7.0 in a volume of 150 μl.

For single-point activity assay, OAS and enzyme concentrations were fixed to 5 mM and 100 ng, respectively. The CS-to-substrate ratio is in the substrate saturating range, and therefore, CS is expected to catalyze with maximal velocity. Steady-state kinetics experiments were performed in triplicates with enzyme concentration fixed at 100 ng and OAS concentration varied from 0.1 mM to 8.0 mM. The Na_2_S concentration was fixed at 3 mM in both assays. Substrates were added to buffer and mixed before the reaction was initiated by the addition of CS/mutants. The reaction was allowed to proceed at 30 °C for 20 min. The reaction was terminated by addition of 5% TCA, centrifuged at 13,000 rpm, and 125 μl of supernatant was transferred to a new tube. To this tube 125 μl of glacial acetic acid and 125 μl of acid ninhydrin reagent (250 mg of ninhydrin dissolved in 2 ml of conc. HCl and 3 ml of glacial acetic acid) were added. After mixing, samples were boiled for 10 min at 99 °C in preheated water bath. Samples were cooled to room temperature and diluted with 625 μl of chilled 95% ethanol. Absorbance was recorded at 560 nm, and amount of cysteine produced was calculated from standard curve, which was estimated for various concentrations of cysteine. Initial velocities determined from triplicate steady-state kinetics experiments were fit to Michaelis–Menten model (Equation 1) using nonlinear least squares method.

(1) $$\text{V}=\text{Vmax}*\left[\text{S}\right]/\left(\left[\text{S}\right]+{\text{K}}_{\text{M}}\right)$$

where v is velocity at given substrate concentration, [S] is substrate concentration, V_max_, is maximum velocity at [E]_T_ << [S]_T_ K_M_, is apparent substrate affinity. The turnover rate, k_cat_, is calculated by normalizing V_max_ by [E]_T_, total enzyme concentration. Errors of kinetic parameters were estimated and reported with 95% confidence intervals (1.98∗standard error). To calculate, the catalytic efficiency (k_cat_/K_M_) of M92A and M120A mutants, we divided k_cat_ value of each mutant by average of K_M_, calculated from K_M_ of single and double mutants of M92A and M120A. For wild-type and M96A, k_cat_ of each mutant was normalized with corresponding K_M_ of that enzyme. Errors for k_cat_/K_M_ were calculated by propagating errors associated with k_cat_ and K_M_ using Equation 2.

(2) $$\Delta {\text{k}}_{\text{cat}}/{\text{K}}_{\text{M}}=\left({\text{k}}_{\text{cat}}/{\text{K}}_{\text{M}}\right)*\left(\sqrt{{\left(\Delta kcat/kcat\right)}^{2}+{\left(\Delta Km/Km\right)}^{2}}\right)$$

where Δk_cat_/K_M_, error of catalytic efficiency, is expressed as square root of sum of squares of relative errors of k_cat_ and K_M._ Δkcat and ΔKm are errors estimated with 95% confidence intervals. Further, we compared the significance of differences of catalytic turnover between wild-type and mutants within each group by estimating *p*-values (0.05).

### Absorption spectroscopic analyses of α-aminoacrylate formation

UV-visible spectra of *HiCS*, *StCS*, *MtCS*, and their mutants in complex with the OAS were recorded with Agilent cary-win UV spectrophotometer for estimation of α-aminoacrylate reaction intermediate formation. The spectra were recorded in the buffer with 50 mM Tris-Cl (pH 7.5) 100 mM NaCl, at 25 °C, and 5% glycerol. Concentrations of *Hi*CS and its mutants used were ∼ 8 to 15 μM and for *St*CS/*M*tCS and its mutants were ∼ 8 to 25.0 μM. The spectra of the covalent α-amino acrylate complex were obtained after the addition of 100 μM OAS to the enzyme solution. Each enzyme was added to the above buffer, mixed, and OAS dissolved in the same buffer was added and mixed to the final volume of ∼800 μl. PLP absorbs at 412 nm. Addition of OAS to CS results in the formation of intermediates with new absorption spectra at 321 nm and 470 nm.

### Equilibrium measurements of OAS and peptide binding to CS and mutants

Both OAS and SAT-C10 peptide binding to CS and its mutants were examined by monitoring fluorescence changes of the active site PLP. The excitation wavelength was set at 412 nm and fluorescence was monitored at 507 nm. All experiments were done in triplicates and at 23.0 °C ± 1 deg. C with excitation and emission bandpass set to 5.0 nm. For OAS experiments, the enzymes concentration was fixed at 0.2 μM and for peptide binding, enzyme concentration was kept between 0.5 and 1.0 μM, and enzyme solution was mixed after each addition of ligands (OAS/peptides). For OAS quenching experiments, the (F_0_) fluorescence of free enzyme is taken as the reference state, and (F) fluorescence of enzymes is taken in the presence of OAS. Initial fluorescence data of wild-type and mutants at a fixed enzyme concentration (0.2 μM) are shown in absolute fluorescence intensity units (Fig. S8). We performed exploratory experiments to determine the saturating OAS concentration and time required for saturation. Briefly, using *Hi*CS as the reference, we monitored the quenching of PLP fluorescence as a function of OAS concentration after incubating for 15 min. Upon estimating the OAS concentration to achieve the maximum quenching, we performed another set of scouting experiments to determine the minimal equilibration time. We fixed CS and OAS concentrations (0.2 μM and 2.0 μM) and monitored the PLP signal as a function of time and found that PLP quenching saturates at 2 to 3 min of incubation. Fluorescence data at each titration point represents the average of 90 readings (30 for each experiment performed in triplicates), and associated errors were estimated with 95% confidence intervals. We performed all OAS binding experiments for both wild-type and mutants by keeping buffer conditions, concentrations, and incubation time fixed. The ratio of F/F_0_ is plotted *versus* enzyme type, and percentage of quenching is estimated from 1 − F/F_0_ for each enzyme type. Mean values of each measurement were shown with errors estimated at 95% confidence intervals.

In the case of peptide binding experiments, we used stocks of peptide concentration in the range of 0.1 to 0.8 mM. After each addition, the reaction mixture was equilibrated for 2 to 3 min before recording PLP fluorescence, and data points from five such measurements were averaged to obtain F_ave,i_. The relative fluorescence quenching upon ligand binding is defined as F_obs,i_ = (F_ave,I_ − F_o_)/F_o_. Inhibitor–CS complex formation was analyzed to obtain the equilibrium binding constant, K_obs_ = [PL]/[P]∗[L], using two independent site-binding models (Equation 3). The fit parameter K_obs_ was determined with 95% confidence interval.

(3) $$F_{\mathrm{obs}}/F_{\max}=n*\left(K_{\mathrm{obs}}*\mathrm{Lig}\right)/\left(1+K_{\mathrm{obs}}*\mathrm{Lig}\right)$$

Where n is 2, *F*_*obs*_ is observed fluorescence quenching, and *F*_*max*_ is the maximum fluorescence quenching at saturation. Equilibrium dissociation constant (K_d_) was obtained from K_obs_ by taking the inverse K_d_ = 1/K_obs_. Errors were propagated using Equation 4

(4) $$\Delta {\text{K}}_{\text{d}}=1/{\text{K}}_{\text{obs}}*\left(\Delta {\text{K}}_{\text{obs}}/{\text{K}}_{\text{obs}}\right)$$

where Δ K_d_ is error of equilibrium dissociation constant, ΔK_obs_ is error of K_obs_.

### Crystallization, data collection, and structural determination

Mutant proteins were crystallized using sitting drop by vapor diffusion method. A total of 1.0 μl drop containing 10 to 12 mg/ml protein mixed with 1.3 M sodium citrate and 100 mM HEPES pH 7.5 was used for the crystallization. Good-quality crystals were obtained within a week time by incubating at 20 °C. All the mutant crystals were obtained at similar conditions. X-ray data was collected at home source on Rigaku micro focus HF beam equipped with MAR345dtb image plate detector. The data was reduced with HKL2000 software package (45). All the mutant structures were solved using molecular replacement using PHASER (46) using the PDB 4HO1 (wild-type *Hi*CS) as the template. The model building was done with graphics program COOT (47). Structure refinement was performed initially with Refmac (48), followed by Phenix (49) for the final refinement cycles. Any spurious electron density was left unfilled. All the figures were made using Pymol graphic software (50).

### Pre-steady-state kinetics

Pre-steady-state kinetics experiments were performed with Biologic rapid kinetics instrument (SFM400, Biologics, France) equipped with four syringes (10 ml) set in a parallel fashion. Fluorescence data was collected by MOS-250 unit equipped with PMT450 (detector) fitted with long-pass filter (450 nm) (Semrock Inc). All the experiments were done in 20 mM Tris pH 7.5, 20 mM NaCl as the running buffer in the flow lines. All proteins and OAS stocks were dissolved in the same buffer. Samples were excited at 412 nm using slit width of 4 nm, and emission was collected after the passage through emission long-pass filter (450 nm). After initial rapid mixing, time course internal fluorescence intensity data was recorded from the PLP. Protein concentration was used in the range of ∼15 to 30 μM, and OAS concentration was used in the range of (10.0–60.00 μM). However, *Mt*92A and *Mt*96A mutants were excluded due to their high aggregation propensity at higher concentrations. We observed that these two mutants exist as stable dimers below <10.0 μM and show time-dependent aggregation at higher concentrations. Fast kinetic data was collected at millisecond timescale (∼2000 points/s) between 0 and 1 s to capture all fast events that occur before steady state is reached. After 1 s, data is collected at 15 points/s as the late events are generally slow. Four to five traces were averaged and data were analyzed using Biokine analysis program, provided by the manufacturer. The rate of binding (*k*_*obs*_) is obtained by fitting data to single exponential model (Equation 5). The errors estimated for the rates represent two standard deviations with 95% confidence interval.

(5) $$F=A_{0}-A\left[\left(\exp\left(-k_{obs,n}t\right)\right)\right]$$

F is the fluorescence at time t, n is the number of exponential terms, A and k_obs_, are the amplitude and the observed rates, respectively, and A_o_ is the fluorescence intensity at t = 0. k_obs_ values obtained from the fit were plotted against the concentration of ligand. The on-rate (k_on_) and off-rate constants were estimated from slope and intercept of that plot (Equation 6)

(6) $$k_{\mathrm{obs}}=C\left(k_{\mathrm{on}}\right)+k_{\mathrm{off}}$$

## Data availability

All data are included within the article. Coordinates and structure factor amplitudes have been deposited into the Protein Data Bank (http://wwpdb.org) under accession numbers: 5XCP; 7CM8; 5XCN; 5XCW; 7C35. Raw data or further information is available upon request from the corresponding author.

## Conflict of interest

The authors declare that they have no conflict of interest with the contents of this article.
