# Supplementary material for: Molecular mechanism of selective substrate engagement and inhibitor disengagement of cysteine synthase
Source: J Biol Chem. 2020 Nov 24;296:100041. doi: 10.1074/jbc.RA120.014490 (PMC7948407; doi:10.1074/jbc.RA120.014490)
Supplement: Figures S1–S8 [file mmc1.pdf]

Supporting Information

Molecular Mechanism of Selective Substrate Engagement and Inhibitor Dis-engagement of Cysteine Synthase

Abhishek Kaushik, R. Rahisuddin, Neha Saini, Ravi P. Singh, Rajveer Kaur, Sukirte Koul, and S. Kumaran*

Figure S1. Structural view of substrate and inhibitor binding.

Figure S2. Size-exclusion and CD profiles of CS and mutants.

Figure S3. SDS-PAGE analysis for CS and its mutants purified after affinity and size exclusion chromatography.

Figure S4. Effect of Methionine mutations on the cysteine synthesis activity of different CS mutants at saturating substrate concentrations.

Figure S5. Determination of substrate concentration for incubation and equilibration time for fluorescence quenching experiments.

Figure S6. Cartoon view of M120 interactions with substrate/inhibitor binding loop.

Figure S7: Complete pre-steady state kinetics traces of *Hi*M120A and *Hi*M92A proteins.

Figure S8: Raw fluorescence data of wildtype and mutant enzymes.

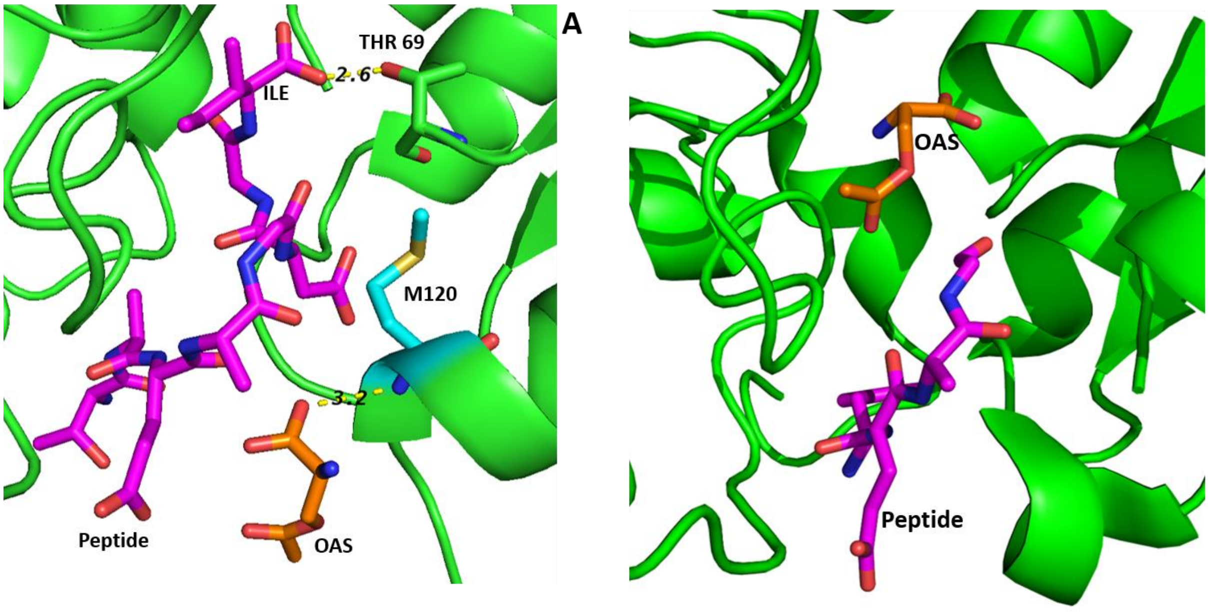

Figure S1. Structural view of substrate and inhibitor binding. A) Ternary complex crystal structure of *Hi*CS with substrate OAS and natural inhibitor peptide (PDB Code: **4ORE**) showed that M120 of α-helix5 is in contact with the in-coming substrate. The C-terminal ILE of inhibitor peptide is anchored to the active site through hydrogen bond between carboxyl oxygen of ILE with side chain of THR69. **B)** Another ternary crystal structure in which the substrate, OAS has entered in to the active site in the presence of bound inhibitor, shows that the crucial hydrogen bonds between the last C-terminal ILE residue of the inhibitor and the "TSGNT" loop broken down and the peptide is forced out (PDB Code: **4ZU6**).

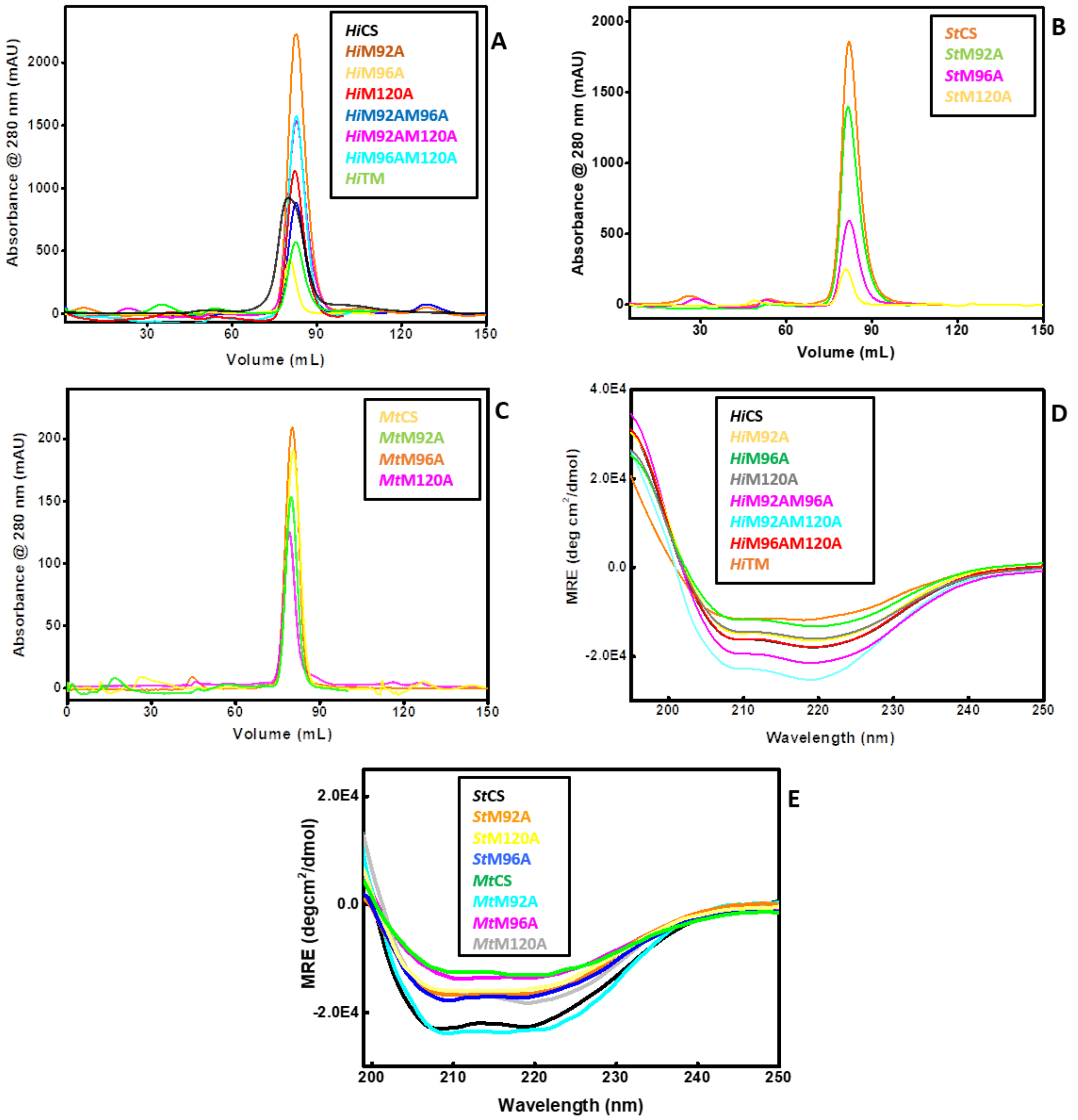

Figure S2. Size-exclusion and CD profiles of CS and mutants. Absorbance at 280 nm versus elution volume plot shows both WT and mutants elute as dimers. A) *Hi*CS and its mutants, eluting at ~ 81 mL. B) *St*CS and its mutants, eluting at ~ 81 mL and C) *Mt*CS and its mutants, eluting at ~ 82 mL. The CD curves for D) *Hi*CS and seven *Hi*CS mutants are shown. E.) *St*CS and its mutants and *Mt*CS and mutants are shown. The protein concentration for CD experiment was used in range of 0.5-2 µM. All the mutant proteins were found to be in dimeric and properly folded state with typical alpha-beta secondary structures.

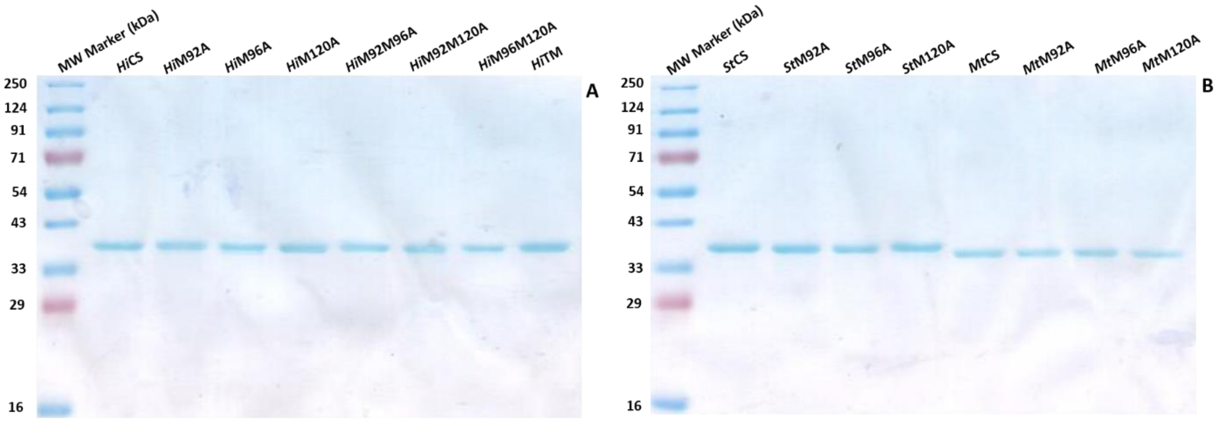

Figure S3. **SDS-PAGE analysis for CS and its mutants purified after affinity and size exclusion chromatography**. 12% SDS-PAGE for A) *Hi*CS and its mutants, B) *St*CS & *Mt*CS and their mutants resolve as a single band showing high homogeneity.

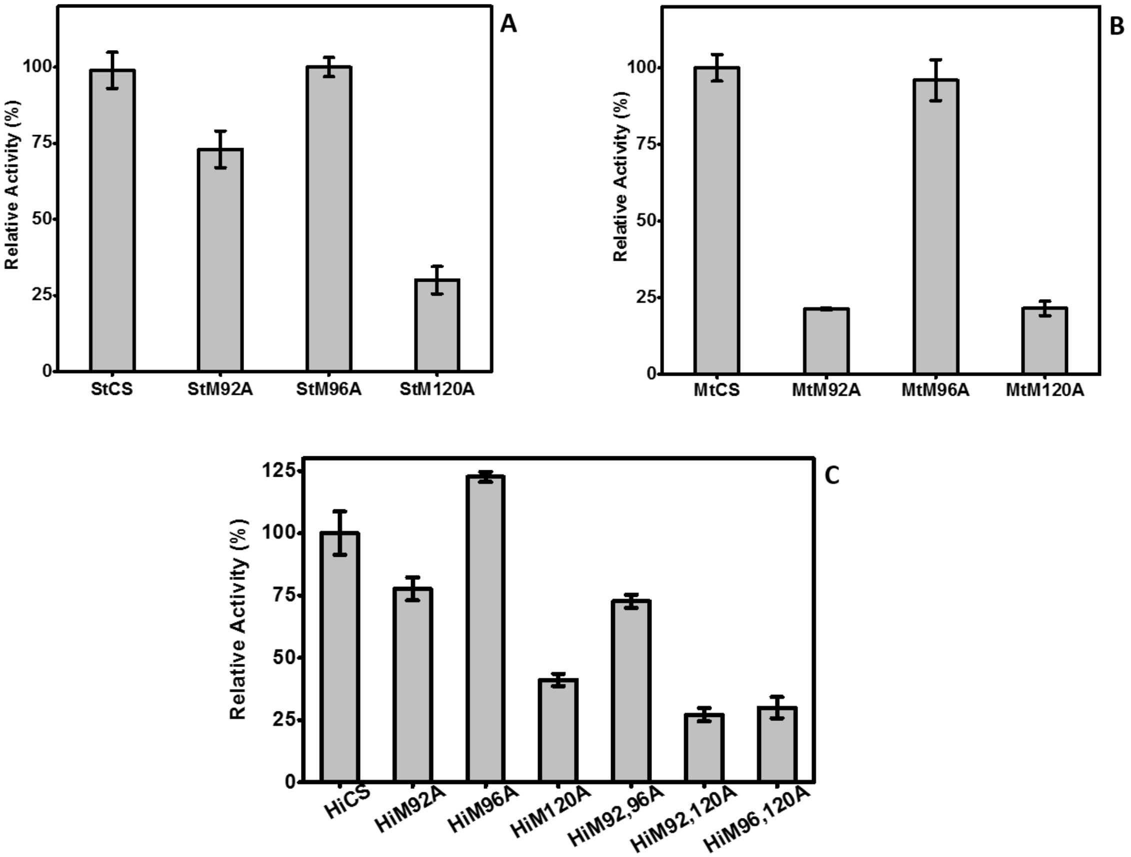

Figure S4. Effect of Methionine mutations on the cysteine synthesis activity of different CS mutants at saturating substrate concentrations. Amounts of cysteine measured from activity assays are normalized with reference to wild type (taken as 100%) plotted are shown. Each error bar represents standard error for single point activity assay that were performed in triplicates. **(A)** Relative cysteine synthesis activities of wildtype and different *StCS* mutants (*St*M92A, *St*M96A and *St*M120A) **(B)** Relative cysteine synthesis activities of wildtype and different *MtCS* mutants (*Mt*M92A, *Mt*M96A and *Mt*M120A). **(C)** Relative cysteine synthesis activities of wildtype and different *HCS* mutants (*Hi*M92A, *Hi*M96A, *Hi*M120A, *Hi*M92M96A, *Hi*M92M120A and *Hi*M96AM120A.

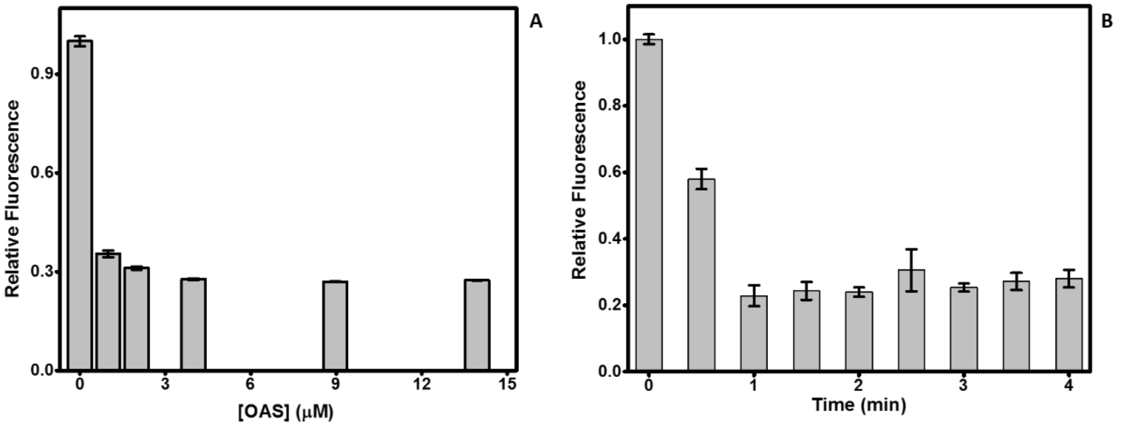

Figure S5. Determination of substrate concentration for incubation and equilibration time for fluorescence quenching experiments. A) PLP fluorescence of *Hi*CS was monitored as function of OAS concentration to choose the minimum concentration of OAS needed for maximum quenching of CS at a fixed protein concentration (0.2 μM). Fluorescence does not change systematically beyond 2.0 μM. Therefore, we fixed OAS concentration at 2.0 μM and protein concentration at 0. 2 μM.. B) PLP fluorescence of *Hi*CS was monitored as a function of time (minutes) at a fixed OAS (2.0 μM) and CS concentration (0.2 μM) to choose the experimental incubation time needed for achieving equilibrium at each titration point. Reading was taken at every 30 seconds till 4 minutes or until no further change in the fluorescence was observed. Result shows no systematic increase in fluorescence quenching after 1-2 minutes and therefore, we chose 2.0 minutes as the mixing and incubation time.

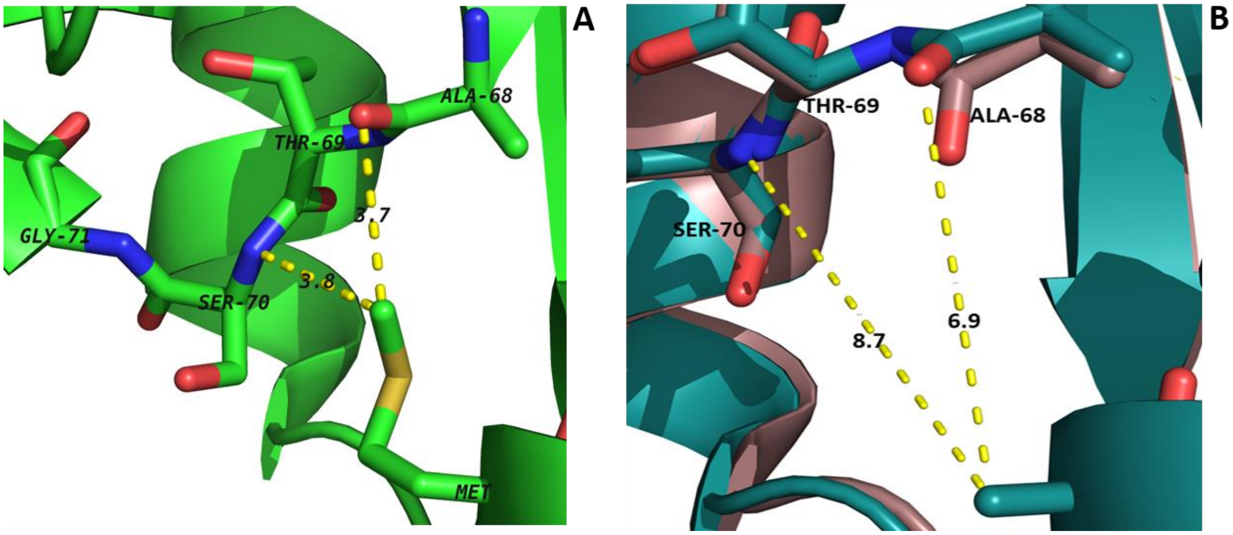

Figure S6. **Cartoon view of M120 interactions with substrate/inhibitor binding loop**. **A)** The interactions between Met120 residue of *Hi*CS to the "TSGNT" loop. Amino group (NH2) of Ser70 interacts with the side chain of Met-120 residue at a distance of 3.8 Å. The main chain carbonyl group of Ala68 makes the interactions with side chain of Met120 residue with the distance of 3.7 Å. **B)** In *Hi*M120A and *Hi*M92AM120A structure, the interaction between main chain carbonyl group of Ala68 and the side chain of Ala120 has been broken and the loop moves to post-inhibitor binding conformation. Also, the Ser70 residue of the loop moves away to the post-inhibitor conformation binding with a distance of 8.7 Å from the Ala120.

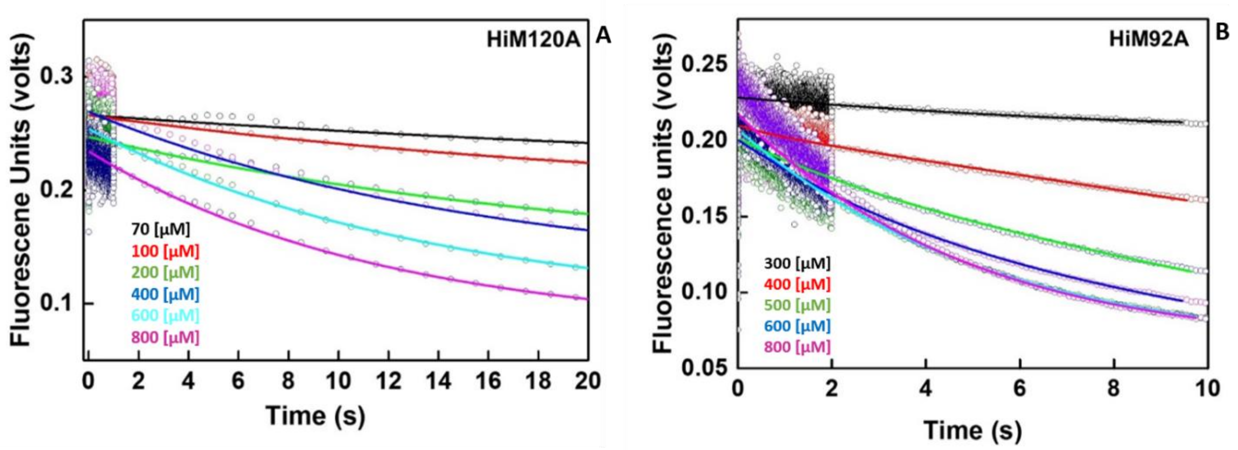

Figure S7: **Complete pre-steady state kinetics traces of *Hi*M120A and *Hi*M92A proteins.**

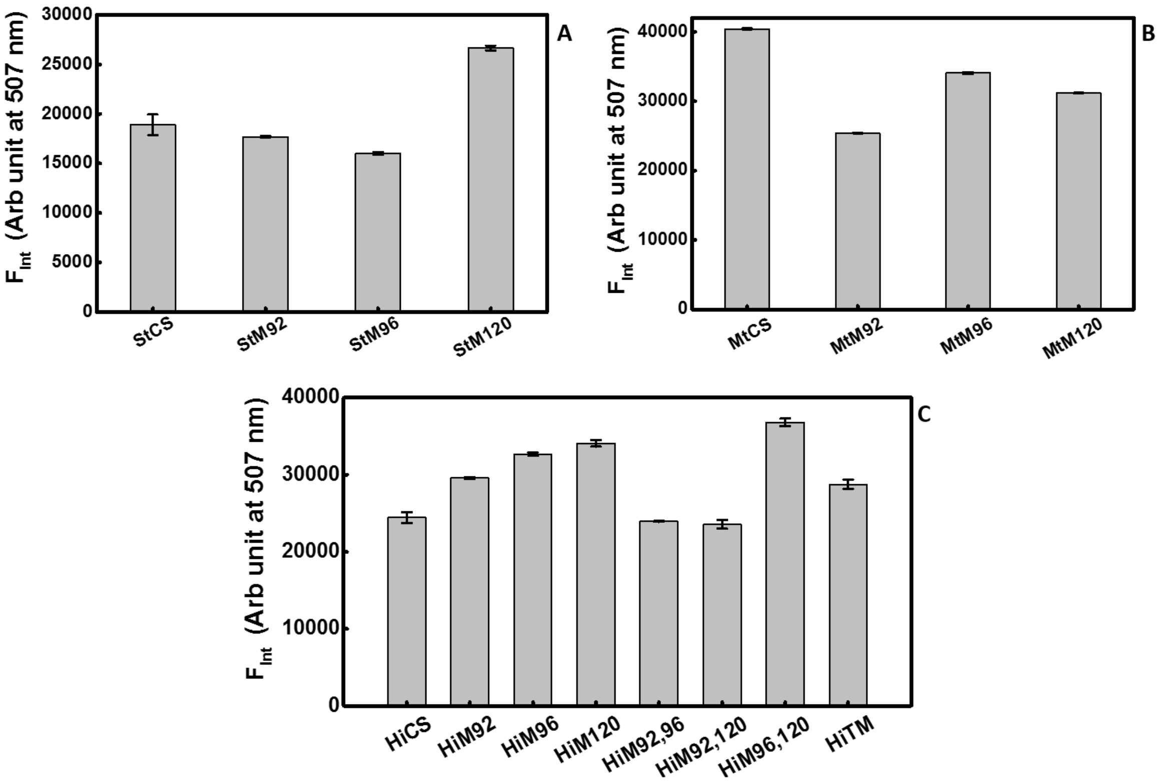

Figure S8: **Raw fluorescence data of wildtype and mutant enzymes**. Arbitrary initial fluorescence intensities plotted against enzyme types. Both excitation and emission bandwidths of fluorometer (Photo Technology International, USA) were fixed at 5 nm. Each Bar with error represents the mean value with standard deviation of 90 data points. A) *St*CS and mutants, B) *Mt*CS and mutants, C) *Hi*CS and mutants.
